# Study on the Anti-*Mycobacterium marinum* Activity of a Series of Marine-Derived 14-Membered Resorcylic Acid Lactone Derivatives

**DOI:** 10.3390/md22030135

**Published:** 2024-03-16

**Authors:** Qian-Qian Jing, Jun-Na Yin, Ya-Jie Cheng, Qun Zhang, Xi-Zhen Cao, Wei-Feng Xu, Chang-Lun Shao, Mei-Yan Wei

**Affiliations:** 1 Key Laboratory of Marine Drugs, The Ministry of Education of China, School of Medicine and Pharmacy, Ocean University of China, Qingdao 266003, China; jingqianqian1231@163.com (Q.-Q.J.); yinjunna@163.com (J.-N.Y.); yajiecheng1212@163.com (Y.-J.C.); zhangqunnn@163.com (Q.Z.); caoxizhen2022@163.com (X.-Z.C.); xuweifeng_u@163.com (W.-F.X.); 2 State Key Laboratory for Chemistry and Molecular Engineering of Medicinal Resources, College of Chemistry and Pharmaceutical Sciences, Guangxi Normal University, Guilin 541004, China; 3 Laoshan Laboratory, Qingdao 266237, China; 4 Key Laboratory of Tropical Medicinal Resource Chemistry of Ministry of Education, College of Chemistry and Chemical Engineering, Hainan Normal University, Haikou 571158, China

**Keywords:** 14-membered resorcylic acid lactones, *Mycobacterium marinum*, *Mycobacterium tuberculosis*, marine natural products, anti-tuberculosis

## Abstract

With the emergence of drug-resistant strains, the treatment of tuberculosis (TB) is becoming more difficult and there is an urgent need to find new anti-TB drugs. *Mycobacterium marinum*, as a model organism of *Mycobacterium tuberculosis*, can be used for the rapid and efficient screening of bioactive compounds. The 14-membered resorcylic acid lactones (RALs) have a wide range of bioactivities such as antibacterial, antifouling and antimalarial activity. In order to further study their bioactivities, we initially constructed a 14-membered RALs library, which contains 16 new derivatives. The anti-*M. marinum* activity was evaluated in vitro. Derivatives **12**, **19**, **20** and **22** exhibited promising activity with MIC_90_ values of 80, 90, 80 and 80 μM, respectively. The preliminary structure–activity relationships showed that the presence of a chlorine atom at C-5 was a key factor to improve activity. Further studies showed that **12** markedly inhibited the survival of *M. marinum* and significantly reduced the dosage of positive drugs isoniazid and rifampicin when combined with them. These results suggest that **12** is a bioactive compound capable of enhancing the potency of existing positive drugs, and its effective properties make it a very useful leads for future drug development in combating TB resistance.

## 1. Introduction

Tuberculosis (TB) is caused by *Mycobacterium tuberculosis* and is one of the deadliest infectious diseases worldwide [1,2]. By 2022, TB became the second leading cause of death globally from a single infectious source after COVID-19, with almost twice as many deaths as HIV/AIDS [3]. Multidrug-resistant tuberculosis (MDR-TB) is a public health crisis and health security threat, and cases of drug-resistant tuberculosis are increasing gradually due to the emergence of multidrug-resistant strains [4,5,6,7]. However, due to the difficulty of anti-tuberculosis drug development and other factors, only bedaquiline, delamanid and pretomanid have been approved for clinical treatment in the past decade, and the first-line drugs are still mainly isoniazid and rifampicin, which were discovered in the 1950s [8,9,10]. Therefore, the development of new anti-TB drugs is crucial.

*M. tuberculosis* is highly infectious and pathogenic, and its culture must be carried out in biosafety level-3 (BSL-3) laboratories [11,12]. Specifically, *M. tuberculosis* grows slowly under in vitro culture conditions, taking about 15 to 20 h to proliferate one generation and 12 days to culture, resulting in a long study cycle. These factors limit the in-depth research of anti-TB drugs [13,14]. In contrast, *Mycobacterium marinum*, from the same genus as *M. tuberculosis*, which grows rapidly with a growth time of about 4 h, is less pathogenic and can be operated in BSL-2 laboratories. More importantly, *M. marinum* and *M. tuberculosis* have high genetic and protein sequence homology. The former’s genome size is 6.5 Mb, which is 2.1 Mb longer than the genes of *M. tuberculosis* [15]. It shares more than 85% of its genome with *M. tuberculosis* and shares major virulence factors. Additionally, *M. marinum* infection in humans usually occurs when broken skin comes into direct contact with infected fish or water sources. After infection, patients exhibit pathological features of TB, such as granuloma. Clinically, *M. marinum* can be effectively treated with anti-TB drugs such as rifampicin, ethambutol and quinolones. Based on the advantages of its good biosafety and ease of operation, *M. marinum* is also widely used as one of the model organisms to study the pathogenesis of *M. tuberculosis* [16,17,18].

Natural products are an important source of new drug development, and more than two-thirds of small molecules approved by the FDA between 1981 and 2019 were related to natural products [19]. The value of natural products of marine origin cannot be ignored. The unique marine environment has created complex, novel and diverse natural products, and endow marine natural products with diversity and particularity in pharmacological activity [20,21]. In terms of anti-TB activity, marine compounds have shown important research value and are valuable resources for the development of anti-TB drugs [22].

**Figure 1:**
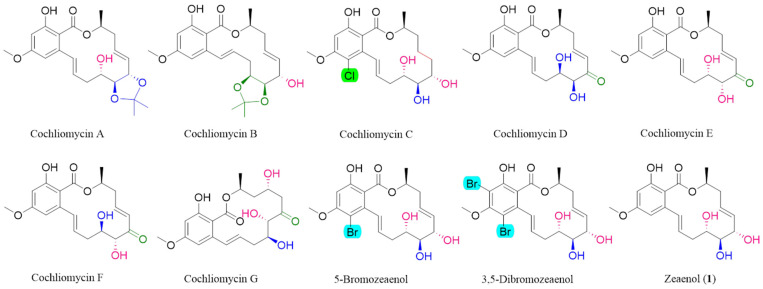
Fourteen-membered lactones isolated from *Cochliobolus lunatus* [23,25,26,27].

The search for new bioactive natural products and derivatives from marine-derived fungi is an ongoing focus of our laboratory. One of our research subjects focuses on resorcylic acid lactones (RALs), polyketide natural products with a 14-membered macrocyclic ring fused to a resorcylic acid residue, which have antibacterial, antifouling, antimalarial and other activities [23,24]. In our previous research, a series of bioactive natural 14-membered RALs (Cochliomycins A–G, 5-Bromozeaenol and 3,5-Dibromozeaenol) were isolated from the marine-derived fungus *Cochliobolus lunatus* (Figure 1) [23,25,26,27]. Especially, cochliomycin A, at a concentration of 1.2 μg/mL, showed significant antifouling activity against the barnacle *Balanus Amphitrite* [23]. In addition, a series of 14-membered RAL derivatives with antiplasmodial and antifouling activities have been discovered [25,26,27,28,29,30].

In this study, we constructed a library consisting of zeaenol (**1**) and its derivatives, **2**–**97**, aimed to enrich the structural diversity of 14-membered RALs and evaluate their structure–activity relationships. Among the synthesized compounds, **19** and **24**–**38** are new derivatives. In addition, six different bacteria and fungi were selected for the in vitro screening of activity. Derivatives **12**, **19**, **20** and **22** have selective activity against *M. marinum*. A further study showed that compound **12** significantly inhibited the survival of *M. marinum*, and the combination with positive drugs significantly reduced the dose of positive drugs isoniazid and rifampicin.

## 2. Results and Discussion

### 2.1. Chemistry

**Scheme 1:**
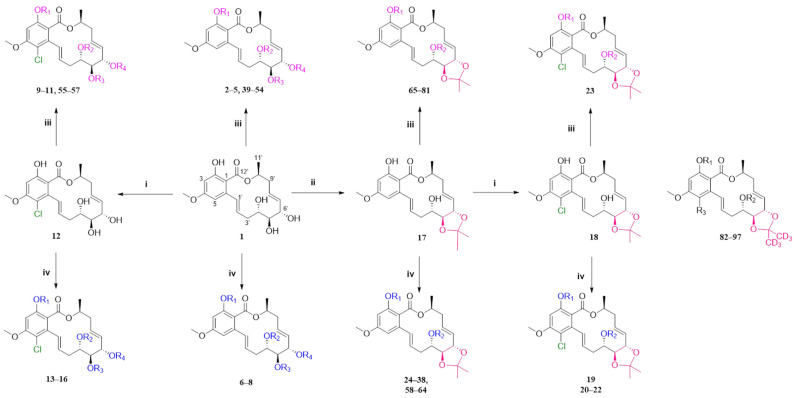
The synthetic route. i SO_2_Cl_2_, 0 °C; ii *p*-TsOH, acetone, room temperature, 5 h; iii ArCH_2_Br reagent, K_2_CO_3_, acetone, 50 °C, 24 h; iv anhydride, acyl chloride reagents or carboxylic acids, DMAP, EDCl, DCM, 45 °C, 3–4 h. Compounds **82**–**97** were synthesized in the same way as other compounds were, except acetone-*d*_6_ was used for acetal formation. The specific information of compounds **2**–**18**, **20**–**23**, **39**–**97** was shown in Ref. [27].

The fermentation condition of *Cochliobolus lunatus* (CHNSCLM-0009) was liquid fermentation. The crude extract obtained after fermentation was subjected to silica gel column chromatography (CC) and recrystallization, and a total of 7.54 g of zeaenol (**1**) was obtained [29,30]. 97 derivatives were semi-synthesized using zeaenol (**1**) as a starting material through one to three steps (Scheme 1). Synthetic schemes for some of the compounds (**2**–**18**, **20**–**23** and **39**–**97**) can be seen in reference [30].

Among the 97 compounds of the library, 38 representative derivatives are shown in Table 1, including the new compounds **19** and **24**–**38**. Compounds **39**–**97** are shown in Table S1.

### 2.2. Evaluation of Biological Activity

#### 2.2.1. Anti-*M. marinum* and Other Antimicrobial Activity

**Figure 2:**
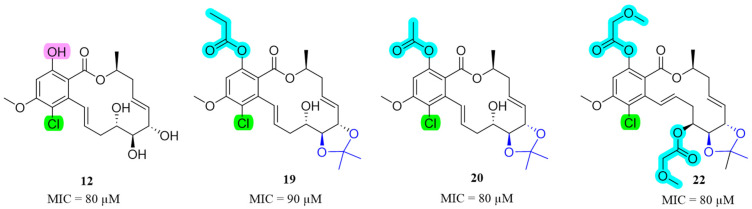
Chemical structures of derivatives **12**, **19**, **20** and **22**, and their anti-*M. marinum* activity.

The cases of drug-resistant tuberculosis are increasing gradually due to the emergence of multidrug-resistant strains [31,32]. As a model organism of *M. tuberculosis*, *M. marinum* can quickly and efficiently screen bioactive compounds. The antibacterial and antifungal activity of the 97 RAL derivatives were evaluated. We discovered several compounds exhibiting potent antibacterial activity, as evidenced by the data presented in Table 2. The activities of the remaining derivatives (MIC_90_ > 200 µM) are not shown in Table 2. Derivatives **12**, **19**, **20** and **22** exhibited promising anti-*M. marinum* activity with MIC_90_ values of 80, 90, 80 and 80 μM, respectively (Figure 2). Comparatively, the MIC_90_ of isoniazid was 40 μM, which indicated the presence of derivatives **12**, **19**, **20** and **22,** and these exhibit good activity. It is worth noting that they also have antibacterial activity selectivity. Compound **1** was the raw material for all derivatives and did not exhibit antimicrobial activity. Derivatives **12**, **19**, **20** and **22** showed substantially improved anti-*M. marinum* activity compared with compound **1**. In order to determine the safety of these compounds, we selected the active derivatives **12**, **19**, **20** and **22** to evaluate non-small cell lung cancer (A549), and the results showed that the IC_50_ values of the above compounds were between 100 and 600 µM, which fully verified that the compounds had own antibacterial effects rather than toxicity.

An overview of the MICs of this 14-membered RAL library in combination with their structures gives preliminary insights of SARs: (1) the anti-*M. marinum* activity of 14-membered RALs can be significantly improved via the introduction of the chlorine atom at the C-5 position, as seen by comparing the MICs of **1**/**12**; (2) bearing an acetonide group at position *5’6’* reduces activity, as seen by comparing the MICs of **12**/**18**; (3) the acetyl- and propanoyl-substituted phenolic hydroxy group enhanced activity when both the chlorine atom, and an acetonide group at *5’6’* were also present (**19**, **20** and **22**); (4) a comparison of compound **12** with compounds **9**–**11** indicates that when the phenolic hydroxy group is transformed into an ether, the activity decreases.

#### 2.2.2. Time−Growth Curves of *M. marinum* Strain Treated with Different Concentrations of Compound **12**

According to the above results, derivatives **12**, **19**, **20** and **22** have selective anti-*M. marinum* activity. Initially, we tested the solubility of the active compounds. In the aqueous solution of 1% DMSO, the solubility of **12** was 0.30 mg/mL, and the solubility of derivatives **19**, **20** and **22** was less than 0.10 mg/mL. Due to the high solubility and strong activity of **12**, it was selected for further study. 

**Figure 3:**
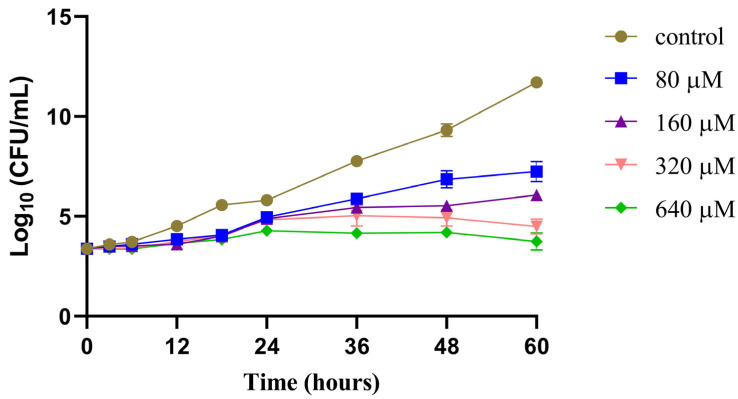
Time−growth curves of *M. marinum* strain treated with different concentrations of compound **12**. Results are expressed as mean ± SEM, *n* = 6.

To investigate its effect on the survival of the *M. marinum* strain, we treated the *M. marinum* strain with different concentrations of compound **12** and plotted a time–growth curve (Figure 3). The results showed that the higher the concentration of compound **12**, the stronger its inhibitory effect on the survival of *M. marinum*, showing a certain concentration dependence. The anti-*M. marinum* effect of **12** began to play a role in 24 h. From that point onwards, the inhibition rate of **12** significantly exceeded the growth rate of the bacteria, resulting in a gradually widening gap between the number of bacteria in the treated group and the control group. By 48 h, a noticeable difference between the groups emerged; however, neither completely eradicated the bacteria.

#### 2.2.3. Anti-*M. marinum* Effects of Compound **12** in Combination with Positive Drugs

**Figure 4:**
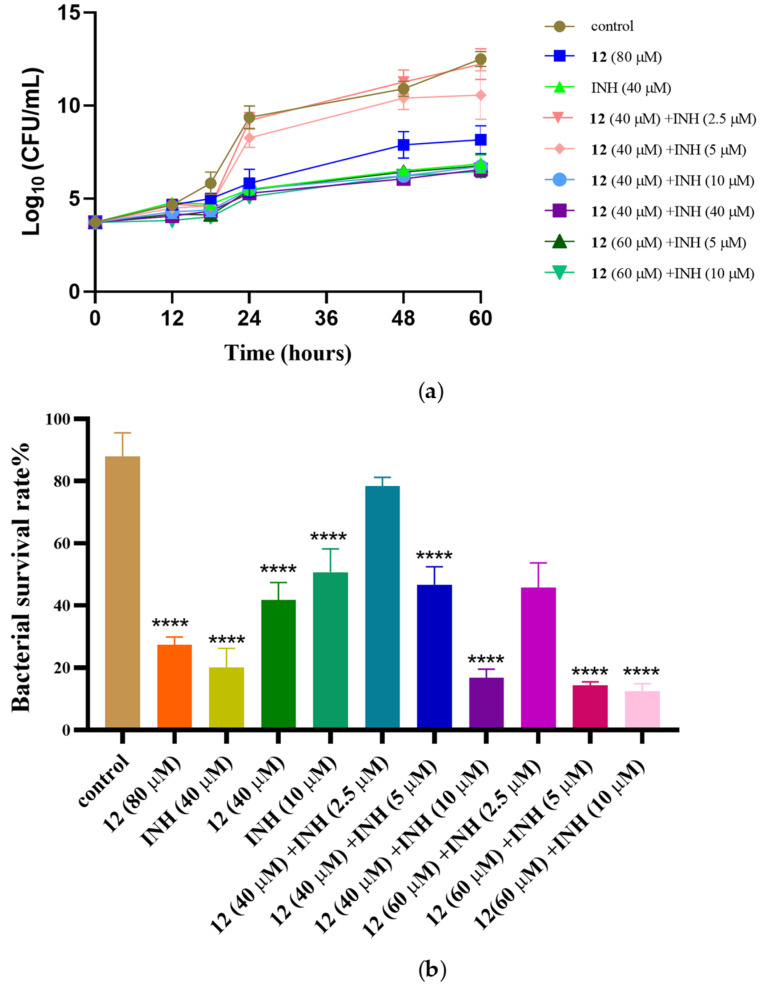
Compound **12** in combination with positive drugs isoniazid (INH). (**a**) Time–growth curves of *M. marinum* strain treated with different concentrations of compound **12** and INH; (**b**) different concentrations of **12** and INH inhibit the growth of *M. marinum*. The number of bacterial cells was measured at 48 h. Data are presented as the mean of three experiments ± SD. **** *p* < 0.0001 compared with the control group.

Current TB treatment regimens are extremely challenging, with about 20% of TB deaths caused by drug-resistant *M. tuberculosis*. MDR-TB is at least resistant to isoniazid and rifampicin, the two most important first-line drugs with which to treat TB. It is necessary to reduce the use of both drugs. We used the checkerboard method [33] to combine **12** with positive drugs for a drug sensitivity test, and the MIC_90_ values for combined medication are shown in Table 3. The results showed that **12** combined with isoniazid and rifampicin had an obvious additive effect. We observed that **12** can significantly reduce the dosage of the positive drugs isoniazid and rifampicin. Compound **12** at 40 μM made *M. marinum* four-fold more sensitive to isoniazid and rifampicin, while 20 μM isoniazid or 2.5 μM rifampicin did not inhibit bacterial growth on average. In particular, when the concentration of **12** was adjusted to 60 μM, *M. marinum* was eight-fold more sensitive to isoniazid and six-fold more sensitive to rifampicin. We selected **12** in combination with isoniazid for bacterial count statistics (Figure 4). It is worth mentioning that **12** did not significantly inhibit bacterial growth in the concentration range of 40–60 μM. Therefore, **12** is considered to be an active compound that can improve the sensitivity of positive drugs. 

## 3. Materials and Methods

### 3.1. General Experimental Procedures

Reagents and solvents were purchased from commercial suppliers and used without further purification. Column chromatography (CC) was performed on silica gel (Qingdao Haiyang Chemical Group Co., Qingdao, China; 200–300 mesh) and Sephadex LH-20 (Amersham Biosciences, Amersham, UK). TLC silica gel plates (Yantai Zifu Chemical Group) were used for thin-layer chromatography. Semi-preparative HPLC was performed on a Waters 1525 system using a C18 column (Amsterdam, The Netherland; Kromasil, 5 μm, 10 × 250 mm) equipped with a Waters 2996 photodiode array detector, and the flow rate was 2.0 mL/min. NMR spectra were recorded on Bruker Advance NEO 400. Chemical shifts, *δ*, were measured in ppm, the internal standard was TMS and coupling constants (*J*) were measured in Hz.

### 3.2. Fungal Material

The fungal strain *Cochliobolus lunatus* (CHNSCLM-0009) was isolated from a piece of tissue from the inner part of the freshly collected gorgonian coral *Dichotella gemmacea* (GX-WZ-20080034), which was collected from the Weizhou coral reef in the South China Sea in September 2008. The fungus was identified as *C. lunatus* via 16*S* rRNA gene analysis, and code ZJ2008002 was obtained. The strain was stored at the Key Laboratory of Marine Drugs, the Ministry of Education of China, School of Medicine and Pharmacy, Ocean University of China, Qingdao, China.

### 3.3. Fermentation, Extraction and Isolation

The fermentation condition of *Cochliobolus lunatus* (CHNSCLM-0009) was liquid fermentation. This fungus was placed in a 500 mL flask with 200 mL of liquid medium (soluble starch 10 g/L, NaNO_3_ 5 g/L, NaOAc 1 g/L, 1% salinity), and fermented on a rotating shaker at 120 r/min at 28 °C for 10 days [29,30]. 

The fermentation liquid was extracted with an equal volume of EtOAc 3–4 times, and concentrated to obtain 25 g of crude extract. The total crude extract was separated via silica gel column chromatography (CC). Ethyl acetate and petroleum ether were selected as eluents. Zeaenol (**1**) was obtained in a 60% ethyl acetate/petroleum ether composition. After repeated recrystallization of the crude product (ethyl acetate/petroleum ether/methanol), a total of 7.54 g of zeaenol (**1**) was obtained.

### 3.4. General Synthetic Methods for Compounds ***2***–***97***

The synthesis of reported compounds (**2**–**18**, **20**–**23** and **39**–**97**) is not described in this article. The synthesis and detailed data of these compounds can be found in reference [30]. Here, we describe in detail the synthetic steps of compound **19** and **24**–**38**.

#### 3.4.1. General Procedure for the Synthesis of **19**

Compound **18** (438.14 µmol, 1 equiv), acetyl chloride (77.99 µmol, 2 equiv), DMAP (753.56 µmol, 3 equiv) and EDCl (753.56 µmol, 3 equiv) in dry CH_2_Cl_2_ (15 mL) were stirred at 45 °C for 2 h, and the reaction process was monitored via TLC. After the reaction was completed, it was quenched with saturated NaHCO_3_ (30 mL) aqueous solution and extracted with CH_2_Cl_2_ (30 mL), and the organic layer was evaporated to dryness, leaving the crude product. The crude product was purified via silica gel CC column chromatography (EtOAc/petroleum ether, 1:5, *v*/*v*) to give derivative **19**.

#### 3.4.2. General Procedure for the Synthesis of **24**–**38**

Compounds **17** (404.18 µmol, 1 equiv), anhydride, acyl chloride or carboxylic acid reagents (5 equiv, Supplementary Table S2), DMAP (753.56 µmol, 3 equiv) and EDCl (753.56 µmol, 3 equiv) in dry CH_2_Cl_2_ (15 mL) were stirred at 45 °C for 4 h, and the reaction process was monitored via TLC. After the reaction was completed, it was quenched with saturated NaHCO_3_ (30 mL) aqueous solution and extracted with CH_2_Cl_2_ (30 mL), and the organic layer was evaporated to dryness, leaving the crude product. Derivatives **24**–**38** were purified via silica gel column chromatography.

#### 3.4.3. Characterization Data of Compounds **19**, and **24**–**38**

The structures of all compounds were identified using NMR data and the HR-ESI-MS spectrum. Compounds **19** and **24**–**38** were new derivatives, the details of which are in the Supplementary Materials. Detailed structural information on other known compounds is not indicated.

Compound **19**, white, solid; yield 70.7%; ${\left[\alpha \right]}_{D}^{20}$–14.6° (*c* 0.05, MeOH); ^1^H NMR (400 MHz, CDCl_3_): *δ* ppm 6.61 (1H, s), 6.50 (1H, dd, *J* = 16.0, 1.9 Hz), 5.95 (1H, dt, *J* = 16.0, 4.6 Hz), 5.78 (1H, ddd, *J* = 16.0, 10.0, 3.8 Hz), 5.67–5.48 (2H, overlapped), 4.51 (1H, t, *J* = 8.4 Hz), 4.12 (1H, m), 3.89 (3H, s), 3.80 (1H, dd, *J* = 8.4, 2.2 Hz), 2.77 (1H, ddd, *J* = 14.6, 6.5, 3.9 Hz), 2.47–2.39 (2H, overlapped), 2.32 (1H, m), 2.28 (3H, s), 1.42 (3H, s), 1.38 (3H, s), 1.30 (3H, d, *J* = 6.3 Hz). ^13^C NMR (100 MHz, CDCl_3_): *δ* 169.0 (C), 165.0 (C), 156.9 (C), 147.7 (C), 136.8 (C), 133.2 (CH), 131.4 (CH), 128.9 (CH), 127.1 (CH), 119.7 (C), 119.4 (CH), 108.5 (CH), 105.4 (CH), 81.2 (CH), 75.5 (CH), 69.2 (CH), 67.9 (CH), 56.5(CH_2_), 37.0 (CH_3_), 35.5 (CH_2_), 26.9(CH_3_ × 2), 26.9(CH_3_ × 2), 20.8 (CH_3_), 20.7 (CH_3_). HRESIMS *m*/*z* 479.1480 [M − H]^−^ (calcd for C_24_H_28_O_8_Cl^−^, 479.1467).

Compound **24**, White, solid; yield 83.2%; ${\left[\alpha \right]}_{D}^{20}$–23.6° (*c* 0.1, MeOH); ^1^H NMR (400 MHz, CDCl_3_) *δ* 8.05 (1H, dd, *J* = 7.8, 1.8 Hz), 7.53 (1H, ddd, *J* = 8.4, 7.4, 1.8 Hz), 7.10–6.99 (3H, overlapped), 6.81 (1H, d, *J* = 2.5 Hz), 6.70 (1H, d, *J* = 2.5 Hz), 5.98 (1H, dt, *J* = 15.8, 5.1 Hz), 5.83 (1H, ddd, *J* = 15.1, 10.8, 3.9 Hz), 5.57–5.37 (2H, overlapped), 4.54 (1H, t, *J* = 8.4 Hz), 4.16(1H, m), 3.94–3.86 (4H, overlapped), 3.83 (3H, s), 2.67 (1H, dtd, *J* = 14.0, 4.1, 2.2 Hz), 2.57–2.41 (3H, overlapped), 2.29 (1H, ddd, *J* = 13.9, 12.0, 10.6 Hz), 1.43 (3H, s), 1.34 (6H, m).^13^C NMR (100 MHz, CDCl_3_) *δ* 165.6 (C), 164.1 (C), 161.9 (C), 160.5 (C), 151.6 (C), 139.9 (C), 134.8 (CH), 133.3 (CH), 133.0 (CH), 131.8 (CH), 130.7 (CH), 128.4 (CH), 120.7 (C), 119.1 (CH), 117.9 (C), 112.6 (CH), 110.7 (C), 108.9 (CH), 108.5 (CH), 81.5 (CH), 76.2 (CH), 69.8 (CH), 67.0 (CH), 56.5 (CH_2_), 56.1 (CH_3_), 37.5 (CH_3_), 36.4 (CH_2_), 27.4 (CH_3_ × 2), 20.5 (CH_3_). HRESIMS *m*/*z* 539.2273 [M + H]^+^ (calcd for C_30_H_35_O_9_^+^, 539.2276).

Compound **25**, White, solid; yield 70.3%; ${\left[\alpha \right]}_{D}^{20}$–21.4° (*c* 0.1, MeOH); ^1^H NMR (400 MHz, CDCl_3_) *δ* 7.66 (1H, dd, *J* = 1.7, 0.8 Hz), 7.37 (1H, dd, *J* = 3.5, 0.9 Hz), 7.05 (1H, dd, *J* = 15.6, 2.0 Hz), 6.82 (1H, d, *J* = 2.5 Hz), 6.68 (1H, d, *J* = 2.5 Hz), 6.58 (1H, dd, *J* = 3.5, 1.7 Hz), 5.98 (1H, dt, *J* = 15.7, 5.2 Hz), 5.84 (1H, ddd, *J* = 15.1, 10.8, 3.9 Hz), 5.49 (1H, ddt, *J* = 15.7, 8.9, 1.8 Hz), 5.39 (1H, pd, *J* = 6.4, 3.8 Hz), 4.54 (1H, t, *J* = 8.4 Hz), 4.16 (1H, ddd, *J* = 12.2, 4.3, 2.3 Hz), 3.88 (1H, dd, *J* = 8.0, 2.3 Hz), 3.83 (3H, s), 2.67 (1H, dtd, *J* = 14.0, 4.3, 2.3 Hz), 2.59–2.46 (3H, overlapped), 2.29 (1H, ddd, *J* = 14.0, 12.0, 10.6 Hz), 1.42 (3H, s), 1.36–1.31 (6H, overlapped).^13^C NMR (100 MHz, CDCl_3_) *δ* 165.1 (C), 161.7 (C), 156.7 (C), 150.5 (C), 147.4 (CH), 143.9 (C), 139.9 (C), 132.8 (CH), 131.4 (CH), 130.6 (CH), 128.4 (CH), 119.9 (C), 117.3 (CH), 112.4 (C), 110.6 (CH), 108.6 (CH), 108.0 (CH), 81.2 (CH), 75.9 (CH), 69.7 (CH), 68.6 (CH), 55.8 (CH_2_), 37.1 (CH_3_), 36.1 (CH_2_), 27.1 (CH_3_), 27.0 (CH_3_), 20.1 (CH_3_). HRESIMS *m*/*z* 499.1968 [M + H]^+^ (calcd for C_27_H_31_O_9_^+^, 499.1963).

Compound **26**, White, solid; yield 60.6%; ${\left[\alpha \right]}_{D}^{20}$–17.2° (*c* 0.1, MeOH); ^1^H NMR (400 MHz, CDCl_3_) *δ* 7.67 (1H, dd, *J* = 1.8, 0.8 Hz), 7.60 (1H, dd, *J* = 1.8, 0.8 Hz), 7.38 (1H, dd, *J* = 3.5, 0.9 Hz), 7.23 (1H, dd, *J* = 3.5, 0.9 Hz), 7.12 (1H, dd, *J* = 15.5, 1.9 Hz), 6.84 (1H, d, *J* = 2.5 Hz), 6.70 (1H, d, *J* = 2.5 Hz), 6.59 (1H, dd, *J* = 3.5, 1.7 Hz), 6.53 (1H, dd, *J* = 3.5, 1.7 Hz), 6.06 (1H, dt, *J* = 15.8, 5.2 Hz), 5.93 (1H, ddd, *J* = 15.1, 10.7, 3.9 Hz), 5.65–5.49 (2H, overlapped), 5.41 (1H, m), 4.73 (1H, t, *J* = 8.2 Hz), 4.06 (1H, dd, *J* = 7.8, 2.3 Hz), 3.85 (3H, s), 2.77 (1H, dtd, *J* = 13.8, 4.3, 2.2 Hz), 2.55–2.40 (3H, overlapped), 1.34 (6H, overlapped), 1.26 (3H, s).^13^C NMR (100 MHz, CDCl_3_) *δ* 165.0 (C), 161.8 (C), 158.1 (C), 156.7 (C), 150.6 (C), 147.4 (CH), 146.6 (CH), 144.7 (CH), 143.9 (CH), 139.6 (C), 133.4 (CH), 131.3 (CH), 130.6 (CH), 127.3 (CH), 119.9 (C), 118.3 (CH), 117.3 (CH), 112.4 (C), 112.0 (CH), 110.5 (CH), 109.3 (CH), 108.3 (CH), 79.8 (CH), 70.8 (CH), 69.8 (CH), 55.8 (CH), 37.3 (CH_2_), 35.2 (CH_3_), 29.8 (CH_2_), 27.3 (CH_3_), 26.7 (CH_3_), 20.1 (CH_3_). HRESIMS *m*/*z* 593.2027 [M + H]^+^ (calcd for C_32_H_33_O_11_^+^, 593.2017).

Compound **27**, White, solid; yield 73.4%; ${\left[\alpha \right]}_{D}^{20}$–19.7° (*c* 0.1, MeCN); ^1^H NMR (400 MHz, CDCl_3_) *δ* 11.51 (1H, s), 7.61 (1H, dd, *J* = 1.8, 0.8 Hz), 7.24 (1H, d, *J* = 2.5 Hz), 6.54 (1H, dd, *J* = 3.5, 1.8 Hz), 6.49 (1H, d, *J* = 2.6 Hz), 6.41 (1H, d, *J* = 2.6 Hz), 6.08 (1H, ddd, *J* = 15.3, 7.5, 5.5 Hz), 5.81 (1H, ddd, t, *J* = 15.4, 10.6, 3.1 Hz), 5.71–5.54 (2H, overlapped), 5.45 (1H, m), 4.76 (1H, t, *J* = 8.1 Hz), 4.06 (1H, dd, *J* = 7.8, 2.3 Hz), 3.83 (3H, s), 2.84 (1H, ddt, *J* = 14.4, 5.4, 2.9 Hz), 2.61–2.39 (3H, overlapped), 1.46 (3H, d, *J* = 6.3 Hz), 1.35 (3H, s), 1.26 (3H, s).^13^C NMR (100 MHz, CDCl_3_) *δ* 170.9 (C), 164.9 (C), 164.2 (C), 158.1 (C), 146.7 (CH), 144.7 (CH), 141.9 (C), 134.7 (CH), 132.5 (CH), 129.6 (CH), 125.8 (CH), 118.4 (C), 112.0 (CH), 109.3 (CH), 107.4 (C), 104.5 (CH), 100.6 (CH), 80.3 (CH), 76.2 (CH), 71.0 (CH), 70.9 (CH), 55.6 (CH_2_), 38.1 (CH_3_), 35.1 (CH_2_), 27.4 (CH_3_), 26.7 (CH_3_), 19.6 (CH_3_). HRESIMS *m*/*z* 499.1962 [M + H]^+^ (calcd for C_27_H_31_O_9_^+^, 499.1963).

Compound **28**, White, solid; yield 63.7%; ${\left[\alpha \right]}_{D}^{20}$–38.1° (*c* 0.1, MeOH); ^1^H NMR (400 MHz, CDCl_3_) *δ* 8.20–8.13 (2H, overlapped), 7.63 (1H, m), 7.50 (2H, overlapped), 7.06 (1H, dd, *J* = 17.2, 2.0 Hz), 6.82 (1H, d, *J* = 2.5 Hz), 6.69 (1H, d, *J* = 2.5 Hz), 5.98 (1H, dt, *J* = 15.8, 5.1 Hz), 5.84 (1H, ddd, *J* = 15.2, 10.8, 3.9 Hz), 5.50 (1H, ddt, *J* = 15.7, 8.8, 1.8 Hz), 5.39 (1H, m), 4.54 (1H, t, *J* = 8.4 Hz), 4.17–4.09 (1H, overlapped), 3.89 (1H, dd, *J* = 8.0, 2.3 Hz), 3.84 (3H, s), 2.67 (1H, dtd, *J* = 14.0, 4.1, 2.2 Hz), 2.58–2.44 (3H, overlapped), 2.30 (1H, ddd, *J* = 14.0, 12.0, 10.6 Hz), 1.42 (3H, s), 1.35 (3H, s), 1.30 (3H, d, *J* = 6.3 Hz).^13^C NMR (100 MHz, CDCl_3_) *δ* 165.2 (C), 165.1 (C), 161.7 (C), 151.3 (C), 139.8 (C), 133.8 (CH), 132.7 (CH), 131.4 (CH), 130.4 (CH), 129.4 (C), 128.7 (CH), 128.3 (CH), 117.4 (CH), 110.4 (CH), 108.6 (CH), 108.0 (C), 81.2 (C), 75.9 (CH), 69.5 (CH), 68.6 (CH), 60.5 (CH), 55.8 (CH), 37.1 (CH), 36.1 (CH_2_), 27.1 (CH_3_), 27.1 (CH_2_), 21.2 (CH_3_), 20.1 (CH_3_), 14.3 (CH_3_). HRESIMS *m*/*z* 509.2176 [M + H]^+^ (calcd for C_29_H_33_O_8_^+^, 509.2170).

Compound **29**, White, solid; yield 77.8%; ${\left[\alpha \right]}_{D}^{20}$–8.1° (*c* 0.1, MeCN); ^1^H NMR (400 MHz, CDCl_3_) *δ* 8.21–8.15 (2H, overlapped), 8.12–8.06 (2H, overlapped), 7.61 (2H, m), 7.49 (4H, overlapped), 7.13 (1H, dd, *J* = 15.5, 1.9 Hz), 6.85 (1H, d, *J* = 2.5 Hz), 6.72 (1H, d, *J* = 2.5 Hz), 6.07 (1H, dt, *J* = 15.7, 5.2 Hz), 5.97 (1H, ddd, *J* = 15.1, 10.7, 3.9 Hz), 5.66 (1H, ddd, *J* = 12.3, 4.6, 2.2 Hz), 5.58 (1H, dd, *J* = 15.8, 8.6 Hz), 5.41 (1H, q, *J* = 5.9 Hz), 4.78 (1H, t, *J* = 8.2 Hz), 4.11 (1H, dt, *J* = 7.8, 2.4 Hz), 3.86 (3H, s), 2.78 (1H, m), 2.56–2.43 (3H, overlapped), 1.32–1.31 (6H, overlapped), 1.23 (3H, s).^13^C NMR (100 MHz, CDCl_3_) *δ* 165.9 (C), 165.2 (C), 161.8 (C), 151.4 (C), 139.5 (C), 133.8 (C), 133.4 (CH), 133.3 (CH), 131.2 (CH), 130.7 (C), 130.4 (C), 130.4 (CH), 129.8 (CH), 129.4 (CH), 128.8 (CH×2), 128.6 (CH×2), 127.5 (CH), 117.5 (CH), 110.4 (C), 109.3 (C), 108.3 (CH), 79.9 (CH), 70.8 (CH), 69.7 (CH), 55.8 (CH), 37.3 (CH), 35.3 (CH_2_), 29.9 (CH_3_), 27.2 (CH_2_), 27.0 (CH_3_ × 2), 20.2 (CH_3_). HRESIMS *m*/*z* 613.2433 [M + H]^+^ (calcd for C_36_H_37_O_9_^+^, 613.2433).

Compound **30**, White, solid; yield 83.5%; ${\left[\alpha \right]}_{D}^{20}$–19.3° (*c* 0.1, MeOH); ^1^H NMR (400 MHz, CDCl_3_) *δ* 8.10 (1H, td, *J* = 7.5, 1.9 Hz), 7.59(m, 1H), 7.28 (1H, dd, *J* = 7.7, 1.1Hz), 7.19 (1H, ddd, *J* = 10.8, 8.3, 1.1 Hz), 7.07 (1H, dd, *J* = 15.5, 2.0 Hz), 6.83 (1H, d, *J* = 2.5 Hz), 6.70 (1H, d, *J* = 2.5 Hz), 5.98 (1H, dt, *J* = 15.8, 5.1 Hz), 5.84 (1H, ddd, *J* = 15.2, 10.8, 3.9 Hz), 5.50 (1H, ddt, *J* = 15.6, 8.8, 1.8 Hz), 5.41 (1H, q, *J* = 5.9 Hz), 4.54 (1H, t, *J* = 8.4 Hz), 4.16 (1H, ddd, *J* = 12.3, 4.4, 2.2 Hz), 3.89 (1H, dd, *J* = 8.0, 2.3 Hz), 3.84 (3H, s), 2.68 (1H, dtd, *J* = 14.0, 4.2, 2.3 Hz), 2.56–2.46 (3H, overlapped), 2.30 (1H, ddd, *J* = 14.0, 12.0, 10.6 Hz), 1.42 (3H, s), 1.37–1.31 (6H, overlapped).^13^C NMR (100 MHz, CDCl_3_) *δ* 165.2 (C), 163.8 (C), 162.6 (C), 161.8 (C), 151.1 (C), 139.9 (C), 135.4 (CH), 135.4 (CH), 132.9 (CH), 132.9 (CH), 131.5 (CH), 130.4 (CH), 128.4 (CH), 124.3 (C), 124.3 (C), 117.9 (CH), 117.2 (C), 110.7 (CH), 108.6 (CH), 108.0 (CH), 81.2 (CH), 75.9 (CH), 69.6 (CH), 68.6 (CH_2_), 55.8 (CH_3_), 37.2 (CH_2_), 36.1 (CH_3_), 27.1 (CH_3_), 20.1 (CH_3_). HRESIMS *m*/*z* 527.2080 [M + H]^+^ (calcd for C_29_H_32_O_8_F^+^, 527.2076).

Compound **31**, White, solid; yield 87.2%; ${\left[\alpha \right]}_{D}^{20}$–28.4° (*c* 0.1, MeOH); ^1^H NMR (400 MHz, CDCl_3_) δ 8.12 (1H, td, *J* = 7.5, 1.9 Hz), 8.03 (1H, m), 7.65–7.49 (2H, overlapped), 7.32–7.11 (5H, overlapped), 6.85 (1H, d, *J* = 2.6 Hz), 6.72 (1H, d, *J* = 2.6 Hz), 6.06 (1H, dt, *J* = 15.8, 5.1 Hz), 5.94 (1H, ddd, *J* = 15.1, 10.8, 3.8 Hz), 5.69 (1H, ddd, *J* = 12.4, 4.8, 2.3 Hz), 5.56 (1H, ddt, *J* = 15.6, 8.5, 1.8 Hz), 5.44 (1H, td, *J* = 6.5, 4.5 Hz), 4.76 (1H, t, *J* = 8.3 Hz), 4.08 (1H, dd, *J* = 7.9, 2.3 Hz), 3.86 (3H, s), 2.80 (1H, ddq, *J* = 13.8, 4.3, 2.3 Hz), 2.60–2.43 (3H, overlapped), 1.38–1.31 (6H, overlapped), 1.26 (3H, s).^13^C NMR (100 MHz, CDCl_3_) *δ* 169.2 (C), 165.2 (C), 163.4 (C), 162.6 (C), 161.8 (C), 161.4 (C), 151.1 (C), 139.6 (C), 135.7 (CH), 135.5 (CH), 134.9 (CH), 133.5 (CH), 132.9 (CH), 132.9 (CH), 132.4 (CH), 131.3 (CH), 130.3 (CH), 127.5 (CH), 124.3 (C), 117.4 (C), 117.3 (C), 117.2 (CH), 117.1 (CH), 110.6 (C), 109.1 (CH), 108.2 (CH), 79.7 (CH), 77.0 (CH), 71.2 (CH), 69.7 (CH), 55.8 (CH_2_), 37.3 (CH_3_), 35.2 (CH_2_), 27.2 (CH_3_), 26.7 (CH_3_), 20.2 (CH_3_). HRESIMS *m*/*z* 649.2242 [M + H]^+^ (calcd for C_36_H_35_O_9_F_2_^+^, 649.2244).

Compound **32**, White, solid; yield 73.9%; ${\left[\alpha \right]}_{D}^{20}$–31.5° (*c* 0.1, MeOH); ^1^H NMR (400 MHz, CDCl_3_) *δ* 11.53 (1H, s), 8.01 (1H, td, *J* = 7.5, 1.9 Hz), 7.55 (1H, m), 7.23 (1H, m), 7.17 (1H, ddd, *J* = 10.9, 8.4, 1.1 Hz), 6.49 (1H, d, *J* = 2.6 Hz), 6.42 (1H, d, *J* = 2.6 Hz), 6.06 (1H, ddd, *J* = 15.4, 7.2, 5.6 Hz), 5.82 (1H, ddd, *J* = 15.4, 10.6, 3.1 Hz), 5.72 (1H, ddd, *J* = 12.4, 5.2, 2.2 Hz), 5.61 (1H, ddt, *J* = 15.5, 8.5, 1.5 Hz), 5.44 (1H, qd, *J* = 6.4, 3.6 Hz), 4.77 (1H, t, *J* = 8.1 Hz), 4.07 (1H, dd, *J* = 7.8, 2.2 Hz), 3.83 (3H, s), 2.87 (1H, ddt, *J* = 14.4, 5.4, 2.8 Hz), 2.50 (3H, m), 2.59–2.42 (4H, overlapped), 1.46 (3H, d, *J* = 6.3 Hz), 1.35 (3H, s), 1.27 (3H, s).^13^C NMR (100 MHz, CDCl_3_) *δ* 171.0 (C), 165.0 (C), 164.2 (C), 142.0 (C), 134.9 (C), 134.8 (C), 134.7 (CH), 132.5 (CH), 132.4 (CH), 129.6 (CH), 126.0 (CH), 124.2 (CH), 118.7 (CH), 117.4 (C), 117.1 (C), 109.1 (CH), 107.4 (C), 104.5 (CH), 100.5 (CH), 80.2 (CH), 76.3 (CH), 71.4 (CH), 71.0 (CH), 55.6 (CH_2_), 38.2 (CH_3_), 35.2 (CH_2_), 27.3 (CH_3_), 26.8 (CH_3_), 19.7 (CH_3_). HRESIMS *m*/*z* 527.2077 [M + H]^+^ (calcd for C_29_H_32_O_8_F^+^, 527.2076).

Compound **33**, White, solid; yield 67.7%; ${\left[\alpha \right]}_{D}^{20}$–24.4° (*c* 0.1, MeOH); ^1^H NMR (400 MHz, CDCl_3_) *δ* 9.35 (1H, dd, *J* = 2.2, 0.9 Hz), 8.85 (1H, dd, *J* = 4.9, 1.8 Hz), 8.43 (1H, dt, *J* = 7.9, 2.0 Hz), 7.46 (1H, ddd, *J* = 7.9, 4.9, 0.9 Hz), 7.08 (1H, dd, *J* = 15.5, 2.0 Hz), 6.85 (1H, d, *J* = 2.5 Hz), 6.70 (1H, d, *J* = 2.5 Hz), 5.98 (1H, dt, *J* = 15.7, 5.2 Hz), 5.85 (1H, ddd, *J* = 15.1, 10.8, 3.8 Hz), 5.48 (1H, ddt, *J* = 15.7, 8.9, 1.8 Hz), 5.35 (1H, m), 4.55 (1H, t, *J* = 8.4 Hz), 4.17 (1H, dt, *J* = 12.3, 3.5 Hz), 3.91–3.84 (4H, overlapped), 2.69 (1H, dtd, *J* = 14.0, 4.1, 2.3 Hz), 2.56 (1H, d, *J* = 1.5 Hz), 2.49 (2H, overlapped), 2.30 (1H, m), 1.42 (3H, s), 1.35 (3H, s), 1.31 (3H, d, *J* = 6.4 Hz).^13^C NMR (100 MHz, CDCl_3_) *δ* 165.1 (C), 164.0 (C), 161.9 (C), 154.1 (C), 151.6 (CH), 151.1 (CH), 140.2(C), 137.8 (CH), 132.8 (CH), 131.4 (CH), 130.5 (CH), 128.5 (C), 125.6 (CH), 123.7 (CH), 117.0(C), 110.7 (C), 108.6 (CH), 108.0 (CH), 81.2 (CH), 75.9 (CH), 69.8 (CH), 68.6 (CH), 55.8 (CH_2_), 37.1 (CH_3_), 36.2 (CH_2_), 27.1 (CH_3_ × 2), 20.1 (CH_3_). HRESIMS *m*/*z* 510.2127 [M + H]^+^ (calcd for C_28_H_32_O_8_N^+^, 510.2122).

Compound **34**, White, solid; yield 85.6%; ${\left[\alpha \right]}_{D}^{20}$–10.3° (*c* 0.05, MeOH); ^1^H NMR (400 MHz, CDCl_3_) *δ* 9.36 (1H, dd, *J* = 2.2, 0.9 Hz), 9.29 (1H, dd, *J* = 2.2, 0.9 Hz), 8.85 (1H, dd, *J* = 4.9, 1.8 Hz), 8.81 (1H, dd, *J* = 4.9, 1.8 Hz), 8.44 (1H, dt, *J* = 8.0, 2.0 Hz), 8.35 (1H, dt, *J* = 8.0, 2.0 Hz), 7.45 (2H, dddd, *J* = 16.4, 8.0, 4.9, 0.9 Hz), 7.17 (1H, dd, *J* = 15.5, 1.9 Hz), 6.88 (1H, d, *J* = 2.5 Hz), 6.73 (1H, d, *J* = 2.5 Hz), 6.07 (1H, dt, *J* = 15.7, 5.2 Hz), 5.96 (1H, ddd, *J* = 15.1, 10.7, 3.8 Hz), 5.70 (1H, ddd, *J* = 12.5, 4.7, 2.3 Hz), 5.55 (1H, ddt, *J* = 15.7, 8.7, 1.7 Hz), 5.39 (1H, td, *J* = 6.5, 4.4 Hz), 4.74 (1H, t, *J* = 8.2 Hz), 4.10 (1H, dd, *J* = 7.8, 2.3 Hz), 3.87 (3H, s), 2.80 (1H, dtd, *J* = 13.8, 4.3, 2.3 Hz), 2.59–2.45 (3H, overlapped), 1.33 (6H, overlapped), 1.22 (3H, s).^13^C NMR (100 MHz, CDCl_3_) *δ* 165.1 (C), 164.6 (C), 164.0 (C), 162.0 (C), 154.2 (C), 153.8 (CH), 151.6 (CH), 151.2 (CH), 151.0 (CH), 139.7 (C), 137.9 (CH), 137.3 (CH), 133.5 (CH), 131.3 (CH), 130.6 (CH), 127.3 (C), 126.2 (C), 125.6 (CH), 123.7 (CH), 123.6 (CH), 117.0 (C), 110.7 (C), 109.3 (CH), 108.4 (CH), 79.7 (CH), 71.3 (CH), 69.9 (CH), 55.9 (CH), 37.2 (CH_2_), 35.2 (CH_3_), 27.2 (CH_2_), 27.0 (CH_3_ × 2), 20.2 (CH_3_). HRESIMS *m*/*z* 615.2318 [M + H]^+^ (calcd for C_34_H_35_O_9_N_2_^+^, 615.2337).

Compound **35**, White, solid; yield 72.4%; ${\left[\alpha \right]}_{D}^{20}$–17.0° (*c* 0.1, MeOH); ^1^H NMR (400 MHz, CDCl_3_) *δ* 11.52 (1H, s), 9.29 (1H, m), 8.81 (1H, d, *J* = 4.0 Hz), 8.35 (1H, dt, *J* = 7.9, 1.9 Hz), 7.44 (1H, dd, *J* = 7.9, 4.8 Hz), 7.25 (1H, d, *J* = 15.4, 2.2Hz), 6.50 (1H, d, *J* = 2.6 Hz), 6.42 (1H, d, *J* = 2.6 Hz), 6.09 (1H, ddd, *J* = 15.3, 7.6, 5.5 Hz), 5.83 (1H, ddd, *J* = 15.4, 10.5, 3.1 Hz), 5.74 (1H, m), 4.77 (1H, t, *J* = 8.0 Hz), 4.11 (1H, dd, *J* = 7.7, 2.3 Hz), 3.83 (3H, s), 2.86 (1H, ddt, *J* = 14.4, 5.5, 2.9 Hz), 2.60–2.44 (3H, overlapped), 1.47 (3H, d, *J* = 6.3 Hz), 1.35 (3H, s), 1.23 (3H, s).^13^C NMR (100 MHz, CDCl_3_) *δ* 170.9 (C), 165.0 (C), 164.6 (C), 164.2 (C), 153.8 (CH), 151.0 (CH), 141.8 (C), 137.3 (CH), 134.8 (CH), 132.6 (CH), 129.7 (CH), 125.6 (C), 123.6 (CH), 109.3 (CH), 107.5 (C), 104.5 (C), 100.6 (CH), 80.2 (CH), 77.4 (CH), 76.4 (CH), 71.5 (CH), 70.9 (CH), 55.6 (CH_2_), 38.1 (CH_3_), 35.2 (CH_2_), 27.2 (CH_3_), 27.0 (CH_3_), 19.6 (CH_3_). HRESIMS *m*/*z* 510.2126 [M + H]^+^ (calcd for C_28_H_32_O_8_N^+^, 510.2122).

Compound **36**, White, solid; yield 63.7%; ${\left[\alpha \right]}_{D}^{20}$–24.6° (*c* 0.1, MeOH); ^1^H NMR (400 MHz, CDCl_3_) *δ* 7.05 (1H, dd, *J* = 15.6, 2.1 Hz), 6.79 (1H, d, *J* = 2.5 Hz), 6.55 (1H, d, *J* = 2.5 Hz), 5.99 (1H, dt, *J* = 15.7, 5.3 Hz), 5.81 (1H, ddd, *J* = 15.5, 10.8, 3.8 Hz), 5.56–5.34 (2H, overlapped), 4.55 (1H, t, *J* = 8.4 Hz), 4.16 (1H, ddd, *J* = 12.3, 4.5, 2.4 Hz), 3.93–3.79 (4H, overlapped), 2.67 (1H, dtd, *J* = 14.0, 4.1, 2.3 Hz), 2.57–2.44 (3H, overlapped), 2.29 (4H, s), 1.39 (9H, m).^13^C NMR (100 MHz, CDCl_3_) *δ* 169.7 (C), 165.2 (C), 161.8 (C), 151.4 (C), 140.0 (C), 132.9 (CH), 131.3 (CH), 130.7 (CH), 128.2 (CH), 117.0 (C), 110.4 (C), 108.6 (CH), 108.0 (CH), 81.2 (CH), 75.9 (CH), 69.7 (CH), 68.7 (CH), 55.7 (CH_2_), 37.2 (CH_3_), 36.1 (CH_2_), 27.1 (CH_3_ × 2), 21.1 (CH_3_), 20.0 (CH_3_). HRESIMS *m*/*z* 447.2011 [M + H]^+^ (calcd for C_24_H_31_O_8_^+^, 447.2013).

Compound **37**, White, solid; yield 85.2%; ${\left[\alpha \right]}_{D}^{20}$–28.3° (*c* 0.1, MeOH); ^1^H NMR (400 MHz, CDCl_3_) *δ* 7.97 (1H, dd, *J* = 3.8, 1.3 Hz), 7.66 (1H, dd, *J* = 5.0, 1.3 Hz), 7.17 (1H, dd, *J* = 5.0, 3.8 Hz), 7.06 (1H, dd, *J* = 15.5, 2.0 Hz), 6.82 (1H, d, *J* = 2.5 Hz), 6.71 (1H, d, *J* = 2.5 Hz), 5.98 (1H, dt, *J* = 15.7, 5.1 Hz), 5.84 (1H, ddd, *J* = 15.2, 10.8, 3.9 Hz), 5.55–5.36 (2H, overlapped), 4.54 (1H, t, *J* = 8.5 Hz), 4.15 (1H, ddd, *J* = 12.2, 4.3, 2.3 Hz), 3.88 (1H, dd, *J* = 8.0, 2.3 Hz), 3.84 (3H, s), 2.67 (1H, dtd, *J* = 13.9, 4.1, 2.2 Hz), 2.49 (3H, overlapped), 2.29 (1H, ddd, *J* = 14.1, 12.0, 10.6 Hz), 1.42 (3H, s), 1.40–1.30 (6H, overlapped).^13^C NMR (100 MHz, CDCl_3_) *δ* 165.2 (C), 161.7 (C), 160.4 (C), 150.8 (C), 139.8 (C), 135.1 (C), 133.8 (CH), 132.9 (CH), 132.6 (CH), 131.5 (CH), 130.3 (CH), 128.4 (CH), 128.2 (CH), 117.4 (C), 110.6 (C), 108.6 (CH), 108.0 (CH), 81.2 (CH), 69.6 (CH), 68.6 (CH), 55.8 (CH), 37.2 (CH_2_), 36.1 (CH_3_), 29.8 (CH_2_), 27.1 (CH_3_), 27.1 (CH_3_), 20.2 (CH_3_). HRESIMS *m*/*z* 515.1730 [M + H]^+^ (calcd for C_27_H_31_O_8_S^+^, 515.1734).

Compound **38**, White, solid; yield 87.7%; ${\left[\alpha \right]}_{D}^{20}$–26.7° (*c* 0.1, MeOH); ^1^H NMR (400 MHz, CDCl_3_) *δ* 7.98 (1H, dd, *J* = 3.8, 1.3 Hz), 7.86 (1H, dd, *J* = 3.8, 1.3 Hz), 7.66 (1H, dd, *J* = 5.0, 1.3 Hz), 7.58 (1H, dd, *J* = 5.0, 1.3 Hz), 7.17 (1H, dd, *J* = 5.0, 3.8 Hz), 7.12 (2H, m), 6.83 (1H, d, *J* = 2.5 Hz), 6.72 (1H, d, *J* = 2.5 Hz), 6.06 (1H, dt, *J* = 15.8, 5.0 Hz), 5.93 (1H, ddd, *J* = 15.1, 10.7, 3.9 Hz), 5.63–5.51 (2H, overlapped), 5.44 (1H, td, *J* = 6.5, 4.7 Hz), 4.72 (1H, t, *J* = 8.2 Hz), 4.05 (1H, dd, *J* = 7.9, 2.3 Hz), 3.85 (3H, s), 2.78 (1H, dtd, *J* = 13.6, 4.3, 2.2 Hz), 2.55–2.41 (3H, overlapped), 1.34 (6H, overlapped), 1.27 (3H, s).^13^C NMR (100 MHz, CDCl_3_) *δ* 165.1 (C), 161.8 (C), 161.5 (C), 160.4 (C), 150.9 (C), 139.5 (C), 135.1 (C), 133.9 (C), 133.7 (CH×2), 133.4 (CH), 132.7 (CH), 132.6 (CH), 131.4 (CH), 130.3 (CH), 128.2 (CH), 128.0 (CH), 127.5 (CH), 117.4 (C), 110.5 (C), 109.2 (CH), 108.3 (CH), 79.8 (CH), 71.0 (CH), 69.7 (CH), 55.8 (CH), 37.3 (CH_2_), 35.2 (CH_3_), 27.3 (CH_2_), 27.0 (CH_3_ × 2), 20.3 (CH_3_). HRESIMS *m*/*z* 625.1545 [M + H]^+^ (calcd for C_32_H_33_O_9_S_2_^+^, 625.1561).

### 3.5. Antimicrobial Activity

The methods described by Fromtling et al. were used to evaluate the derivatives’ antibacterial activity [34]. Isoniazid and rifampicin were used as a positive control anti-*M. marinum*, ciprofloxacin as a positive control anti-bacteria, and amphotericin B as a positive control anti-fungi. The strains were cultured in the corresponding medium at 32 °C for 8 h and diluted to 10^5^ CFU/mL using 96-well plates with 2 μL of sample and 198 μL of bacterial solution. Incubation was carried out at 32 °C for 24 h or 48 h, and DMSO was used as a negative control.

### 3.6. Time–Growth Curve Assay

The time–growth curve was determined using the method of Li et al. [35]. Initially, the concentration of *M. marinum* was set at 10^5^ CFU/mL, and 5 groups were chosen, each group tested on 6 times. The drug concentration of the dosing group was set at 640, 320, 160 and 80 μM. An equal amount of DMSO was added to the blank group, and the suspension of *M. marinum* was incubated at 32 °C (100 rpm) by oscillating it. The colony count was determined and counted using the OD600 at the planned time points (0, 3, 6, 12, 18, 24, 36, 48 and 60 h).

### 3.7. In Vitro Synergic Anti-M. marinum Activity Assay

An in vitro synergistic antibacterial assay was performed as described by Li et al., and synergistic activity was evaluated on 96-well plates [33]. Based on the MIC_90_ data of the compound and each positive drug as the design basis, the chessboard dilution method was used to combine 4 × MIC_90_, 2 × MIC_90_, MIC_90_, 1/2 × MIC_90_, 1/4 × MIC_90_, 1/8 × MIC_90_ and 1/16 × MIC_90_ in the 96-well plate with bacterial solution added. The measurements were repeated three times per well. The 96-well plates were placed in a constant-temperature incubator at 32 °C, the results were observed and recorded 48 h later, and an optical density of 600 nm (OD600) was measured.

### 3.8. Statistical Analysis

All statistical analyses were performed using GraphPad Prism 8.3 software. Data are presented as the mean of three experiments. For two-group comparison, the *p* value was derived from a one-way Student *t* test to determine the difference between groups with normally distributed data. For all comparisons, *p* < 0.05 was considered statistically significant. * *p* < 0.05, ** *p* < 0.01, *** *p* < 0.001 and **** *p* < 0.0001.

## 4. Conclusions

In summary, 16 new derivatives were successfully synthesized through two to three steps, which enriched the diversity of 14-membered RALs. Through the activity evaluation of the derivative library, four derivatives showed promising anti-*M. marinum* activity. The preliminary structure–activity relationships showed that the anti-*M. marinum* activity of 14-membered RALs can be significantly improved via the introduction of a chlorine atom at the C-5 position. The substitution of positions *5’6’* of dihydroxy with an acetonide group reduced the activity. The etherification modification of the phenolic hydroxy group did not significantly improve the activity. Further studies showed that compound **12** enhanced the effects of positive drugs isoniazid and rifampicin on *M. marinum.* These results suggest that **12** is an active compound capable of enhancing the potency of existing positive drugs, and its effective properties make it a very useful lead for future drug development in combating TB resistance.

## Figures and Tables

**Table 1:** Zeaenol (**1**) and its derivatives **2**–**38**.

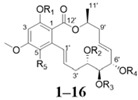											
**No.**	**R_1_**	**R_2_**	**R_3_**	**R_4_**	**R_5_**	**No.**	**R_1_**	**R_2_**	**R_3_**	**R_4_**	**R_5_**
1	H	H	H	H	H	9	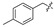	H	H	H	Cl
2	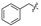	H	H	H	H	10	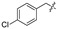	H	H	H	Cl
3	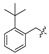	H	H	H	H	11	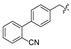	H	H	H	Cl
4	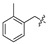	H	H	H	H	12	H	H	H	H	Cl
5	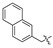	H	H	H	H	13	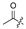	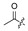	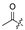	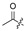	Cl
6	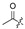	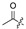	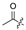	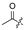	H	14	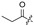	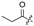	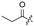	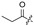	Cl
7	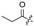	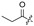	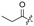	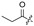	H	15	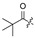	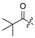	H	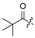	Cl
8	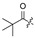	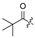	H	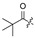	H	16	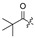	H	H	H	Cl
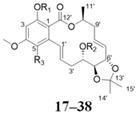											
**No.**	**R_1_**	**R_2_**		**R_3_**		**No.**	**R_1_**	**R_2_**		**R_3_**	
17	H	H		H		28	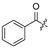	H		H	
18	H	H		Cl		29	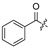	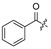		H	
19	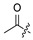	H		Cl		30	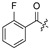	H		H	
20	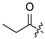	H		Cl		31	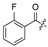	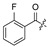		H	
21	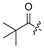	H		Cl		32	H	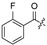		H	
22	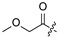	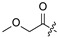		Cl		33	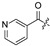	H		H	
23	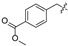	H		Cl		34	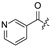	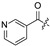		H	
24	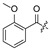	H		H		35	H	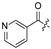		H	
25	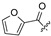	H		H		36	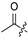	H		H	
26	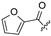	* 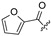 *		H		37	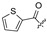	H		H	
27	H	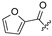		H		38	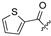	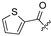		H	

**Table 2:** Antimicrobial activity of representative compounds in the RAL library ^1^.

Compound	MIC_90_ (µM)					
	*M. marinum*	*S. aureus*	*E. coli*	*P*. *aeruginosa*	*C*. *albicans*	*A*. *fumigatus*
**1**	>200	>100	>100	>100	>100	>100
**4**	>200	12.5	>100	>100	>100	>100
**11**	100	>100	>100	>100	>100	>100
**12**	80	>100	>100	>100	>100	>100
**18**	>200	>100	>100	>100	>100	>100
**19**	90	>100	>100	>100	>100	>100
**20**	80	>100	>100	>100	>100	>100
**22**	80	>100	>100	>100	>100	>100
Isoniazid	40	nt	nt	nt	nt	nt
Rifampicin	10	nt	nt	nt	nt	nt
Ciprofloxacin	nt	3.13	0.10	1.56	nt	nt
Amphotericin B	nt	nt	nt	nt	0.84	0.07

^1^ Results are the average of three independent experiments, each performed in duplicate. Standard deviations were less than ±10%. nt = not tested.

**Table 3:** Anti-*M. marinum* effects of compound **12** in combination with positive drugs.

	Positive Drugs MIC_90_ (µM)		Compound 12 MIC_90_ (µM)		FICI ^1^	Mode of Action
	Alone	Combined	Alone	Combined		
Isoniazid	40	10	80	40	0.75	additive
Rifampicin	10	2.5	80	40	0.75	additive

^1^ The mode of action was determined using the fractional inhibitory concentration index (FICI): (1) FICI ≤ 0.5, synergistic effect; (2) 0.5 < FICI ≤ 1, additive effect; (3) 1 < FICI ≤ 2, irrelevant; (4) FICI > 2, antagonistic effect.

## Data Availability

The data are contained within the article or Supplementary Materials.

## Supplementary Materials

The following supporting information can be downloaded at https://www.mdpi.com/article/10.3390/md22030135/s1; Table S1: All compounds that not appear in the text in the derivative library. Table S2: Anhydride, acyl chloride or carboxylic acid reagents used to generate compounds **24**–**38**. Figures S1–S48: ^1^H NMR, ^13^C NMR and HRESIMS of compounds **19** and **24**–**38**.
