# Supplementary material for: Study on the Anti-Mycobacterium marinum Activity of a Series of Marine-Derived 14-Membered Resorcylic Acid Lactone Derivatives
Source: Mar Drugs. 2024 Mar 16;22(3):135. doi: 10.3390/md22030135 (PMC10972006; doi:10.3390/md22030135)
Supplement: Supplementary file 1 [file marinedrugs-22-00135-s001.zip › marinedrugs-2874474-supplementary.pdf]

Study on the Anti-*Mycobacterium marinum* Activity of a Series of Marine-Derived 14-Membered Resorcylic Acid Lactone Derivatives

Qian-Qian Jing [1,†], Jun-Na Yin [1,†], Ya-Jie Cheng [1], Qun Zhang [1], Xi-Zhen Cao [1], Wei-Feng Xu [1,2], Chang-Lun Shao [1,3,4,*], and Mei-Yan Wei [1,*]

[1] Key Laboratory of Marine Drugs, the Ministry of Education of China, School of Medicine and Pharmacy, Ocean University of China, Qingdao 266003, China; jingqianqian1231@163.com (Q.-Q.J.); yinjunna@163.com (J.-N.Y.); yajiecheng1212@163.com (Y.-J.C); zhangqunnn@163.com; (Q.Z.); caoxizhen2022@163.com (X.-Z.C.); xuweifeng_u@163.com (W.-F.X)

[2] State Key Laboratory for Chemistry and Molecular Engineering of Medicinal Resources, College of Chemistry and Pharmaceutical Sciences, Guangxi Normal University, Guilin 541004, China

[3] Laoshan Laboratory, Qingdao 266237, China

[4] Key Laboratory of Tropical Medicinal Resource Chemistry of Ministry of Education, College of Chemistry and Chemical Engineering, Hainan Normal University, Haikou 571158, China

[*] Correspondence: shaochanglun@163.com (C.-L.S); mywei95@126.com (M.-Y.W.)

Content of Supporting Information

Table S1. The derivatives **39 – 97** of zeaenol (**1**).
Table S2. Anhydride, acyl chloride reagents or carboxylic acid reagents used to generate compounds **24–38**.
Figure S1. ^{1}H NMR (400 MHz, Chloroform-*d*) spectrum of compound **19**.
Figure S2. ^{13}C NMR (100 MHz, Chloroform-*d*) spectrum of compound **19**.
Figure S3. HR-ESI-MS spectrum of compound **19**.
Figure S4. ^{1}H NMR (400 MHz, Chloroform-*d*) spectrum of compound **24**.
Figure S5. ^{13}C NMR (100 MHz, Chloroform-*d*) spectrum of compound **24**.
Figure S6. HR-ESI-MS spectrum of compound **24**.
Figure S7. ^{1}H NMR (400 MHz, Chloroform-*d*) spectrum of compound **25**.
Figure S8. ^{13}C NMR (100 MHz, Chloroform-*d*) spectrum of compound **25**.
Figure S9. HR-ESI-MS spectrum of compound **25**.
Figure S10. ^{1}H NMR (400 MHz, Chloroform-*d*) spectrum of compound **26**.
Figure S11. ^{13}C NMR (100 MHz, Chloroform-*d*) spectrum of compound **26**.
Figure S12. HR-ESI-MS spectrum of compound **26**.
Figure S13. ^{1}H NMR (400 MHz, Chloroform-*d*) spectrum of compound **27**.
Figure S14. ^{13}C NMR (100 MHz, Chloroform-*d*) spectrum of compound **27**.
Figure S15. HR-ESI-MS spectrum of compound **27**.
Figure S16. ^{1}H NMR (400 MHz, Chloroform-*d*) spectrum of compound **28**.
Figure S17. ^{13}C NMR (100 MHz, Chloroform-*d*) spectrum of compound **28**.
Figure S18. HR-ESI-MS spectrum of compound **28**.
Figure S19. ^{1}H NMR (400 MHz, Chloroform-*d*) spectrum of compound **29**.
Figure S20. ^{13}C NMR (100 MHz, Chloroform-*d*) spectrum of compound **29**.
Figure S21. HR-ESI-MS spectrum of compound **29**.
Figure S22. ^{1}H NMR (400 MHz, Chloroform-*d*) spectrum of compound **30**.
Figure S23. ^{13}C NMR (100 MHz, Chloroform-*d*) spectrum of compound **30**.
Figure S24. HR-ESI-MS spectrum of compound **30**.
Figure S25. ^{1}H NMR (400 MHz, Chloroform-*d*) spectrum of compound **31**.
Figure S26. ^{13}C NMR (100 MHz, Chloroform-*d*) spectrum of compound **31**.
Figure S27. HR-ESI-MS spectrum of compound **31**.
Figure S28. ^{1}H NMR (400 MHz, Chloroform-*d*) spectrum of compound **32**.
Figure S29. ^{13}C NMR (100 MHz, Chloroform-*d*) spectrum of compound **32**.
Figure S30. HR-ESI-MS spectrum of compound **32**.
Figure S31 ^{1}H NMR (400 MHz, Chloroform-*d*) spectrum of compound **33**.
Figure S32. ^{13}C NMR (100 MHz, Chloroform-*d*) spectrum of compound **33**.
Figure S33. HR-ESI-MS spectrum of compound **33**.
Figure S34. ^{1}H NMR (400 MHz, Chloroform-*d*) spectrum of compound **34**.
Figure S35 ^{13}C NMR (100 MHz, Chloroform-*d*) spectrum of compound **34**.
Figure S36. HR-ESI-MS spectrum of compound **34**.
Figure S37. ^{1}H NMR (400 MHz, Chloroform-*d*) spectrum of compound **35**.
Figure S38. ^{13}C NMR (100 MHz, Chloroform-*d*) spectrum of compound **35**.
Figure S39. HR-ESI-MS spectrum of compound **35**.

Figure S40. ^{1}H NMR (400 MHz, Chloroform-*d*) spectrum of compound **36**.
Figure S41. ^{13}C NMR (100 MHz, Chloroform-*d*) spectrum of compound **36**.
Figure S42. HR-ESI-MS spectrum of compound **36**
Figure S43. ^{1}H NMR (400 MHz, Chloroform-*d*) spectrum of compound **37**.
Figure S44. ^{13}C NMR (100 MHz, Chloroform-*d*) spectrum of compound **37**.
Figure S45. HR-ESI-MS spectrum of compound **37**.
Figure S46. ^{1}H NMR (400 MHz, Chloroform-*d*) spectrum of compound **38**.
Figure S47. ^{13}C NMR (100 MHz, Chloroform-*d*) spectrum of compound **38**.
Figure S48. HR-ESI-MS spectrum of compound **38**.

Table S1. The derivatives **39 – 97** of zeaenol (**1**)

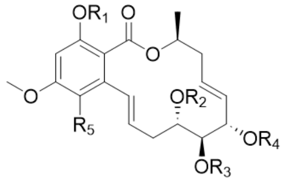

39–57

No.	R1	R2	R3	R4	R5	No.	R1	R2	R3	R4	R5
**39**	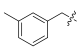	H	H	H	H	**49**	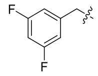	H	H	H	H
**40**	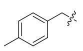	H	H	H	H	**50**	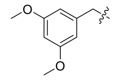	H	H	H	H
**41**	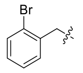	H	H	H	H	**51**	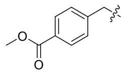	H	H	H	H
**42**	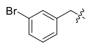	H	H	H	H	**52**	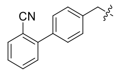	H	H	H	H
**43**	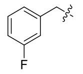	H	H	H	H	**53**	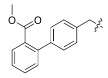	H	H	H	H
**44**	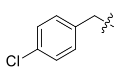	H	H	H	H	**54**	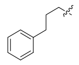	H	H	H	H
**45**	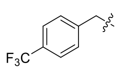	H	H	H	H	**55**	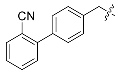	H	H	H	Cl
**46**	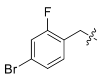	H	H	H	H	**56**	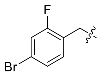	H	H	H	Cl
**47**	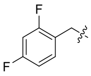	H	H	H	H	**57**	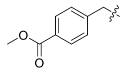	H	H	H	Cl
**48**	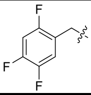	H	H	H	H						

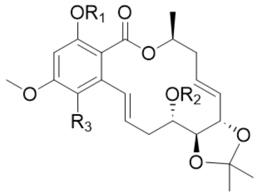

58–81

No.	R1	R2	R3	No.	R1	R2	R3	No.	R1	R2	R3
**58**	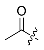	H	H	**66**	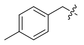	H	H	**74**	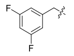	H	H
**59**	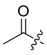	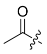	H	**67**	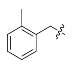	H	H	**75**	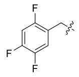	H	H

**60**	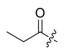	H	H	**68**	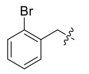	H	H	**76**	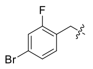	H	H
**61**	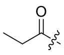	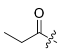	H	**69**	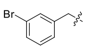	H	H	**77**	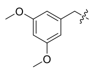	H	H
**62**	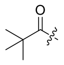	H	H	**70**	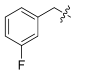	H	H	**78**	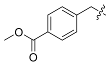	H	H
**63**	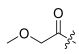	H	H	**71**	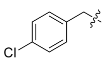	H	H	**79**	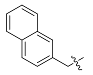	H	H
**64**	H	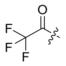	H	**72**	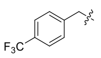	H	H	**80**	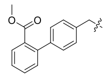	H	H
**65**	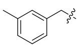	H	H	**73**	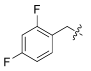	H	H	**81**	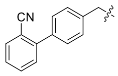	H	H

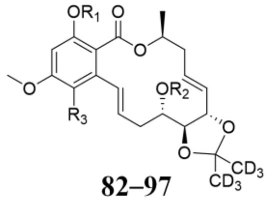

82–97

No.	R1	R2	R3	No.	R1	R2	R3	No.	R1	R2	R3
**82**	H	H	H	**88**	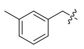	H	H	**93**	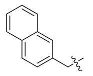	H	H
**83**	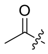	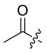	H	**89**	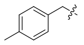	H	H	**94**	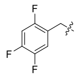	H	H
**84**	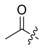	H	Cl	**90**	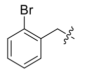	H	H	**95**	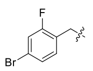	H	H
**85**	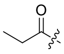	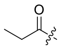	H	**91**	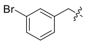	H	H	**96**	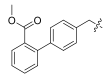	H	H
**86**	H	H	Cl	**92**	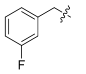	H	H	**97**	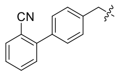	H	H
**87**	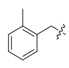	H	H								

Table S2. Anhydride, acyl chloride reagents or carboxylic acid reagents used to generate compounds **24–38**[a].

No.	reagents	Yield	No.	reagents	Yield	No.	reagents	Yield
**24**	2-Methoxybenzoic acid	83.2%	**29**	Benzoyl chloride	77.8%	**34**	Nicotinic acid	85.6%
**25**	2-Furoic acid	70.3%	**30**	2-Fluorobenzoic acid	83.5%	**35**	Nicotinic acid	72.4%
**26**	2-Furoic acid	60.6%	**31**	2-Fluorobenzoic acid	87.2%	**36**	Acetic anhydride	63.7%
**27**	2-Furoic acid	73.4%	**32**	2-Fluorobenzoic acid	73.9%	**37**	Thiophene-2-carboxylic acid	85.2%
**28**	Benzoyl chloride	63.7%	**33**	Nicotinic acid	67.7%	**38**	Thiophene-2-carboxylic acid	87.7%

[a] All reagents used in this study are commercial reagents.

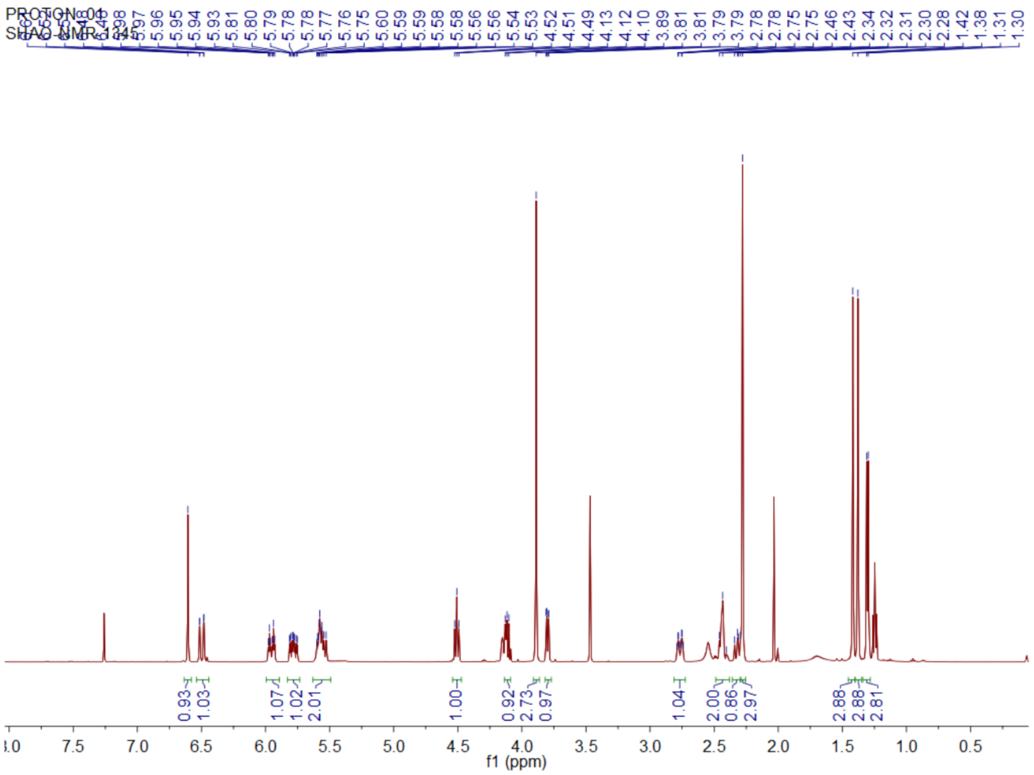

Figure S1. ^{1}H NMR (400 MHz, Chloroform-*d*) spectrum of compound **19**.

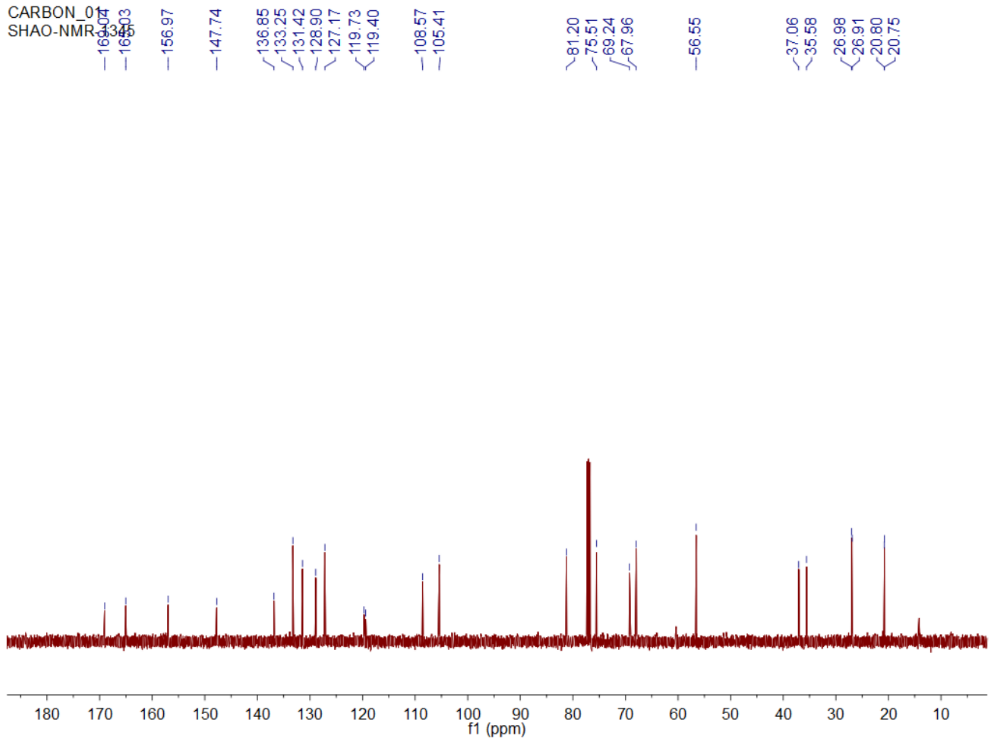

Figure S2. ^{13}C NMR (100 MHz, Chloroform-*d*) spectrum of compound **19**.

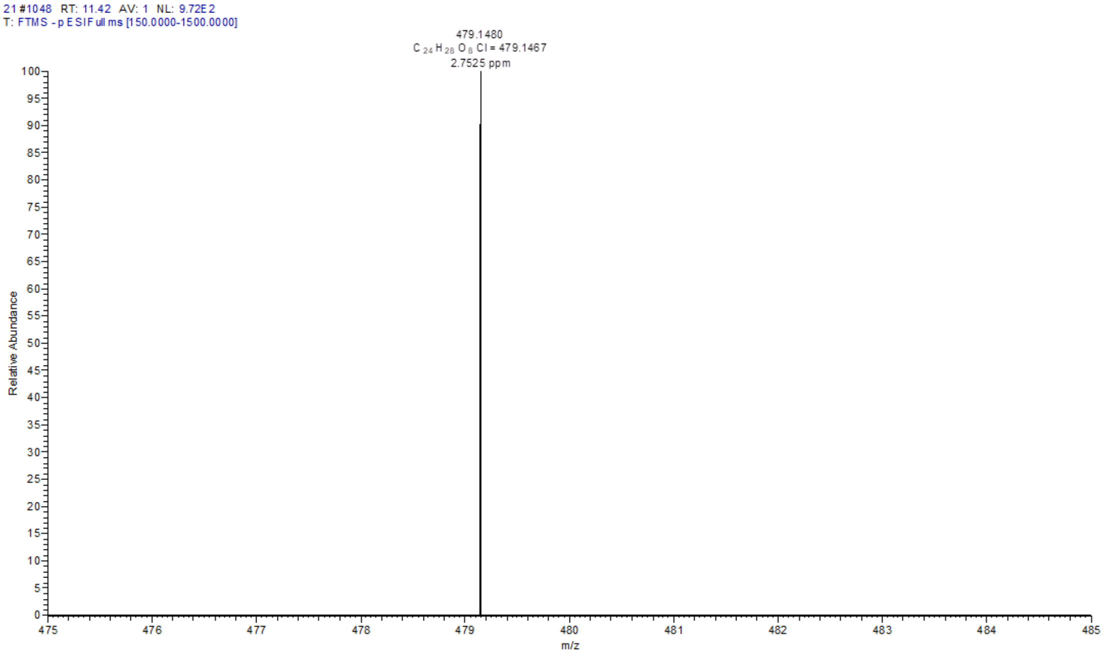

Figure S3. HR-ESI-MS spectrum of compound **19**.

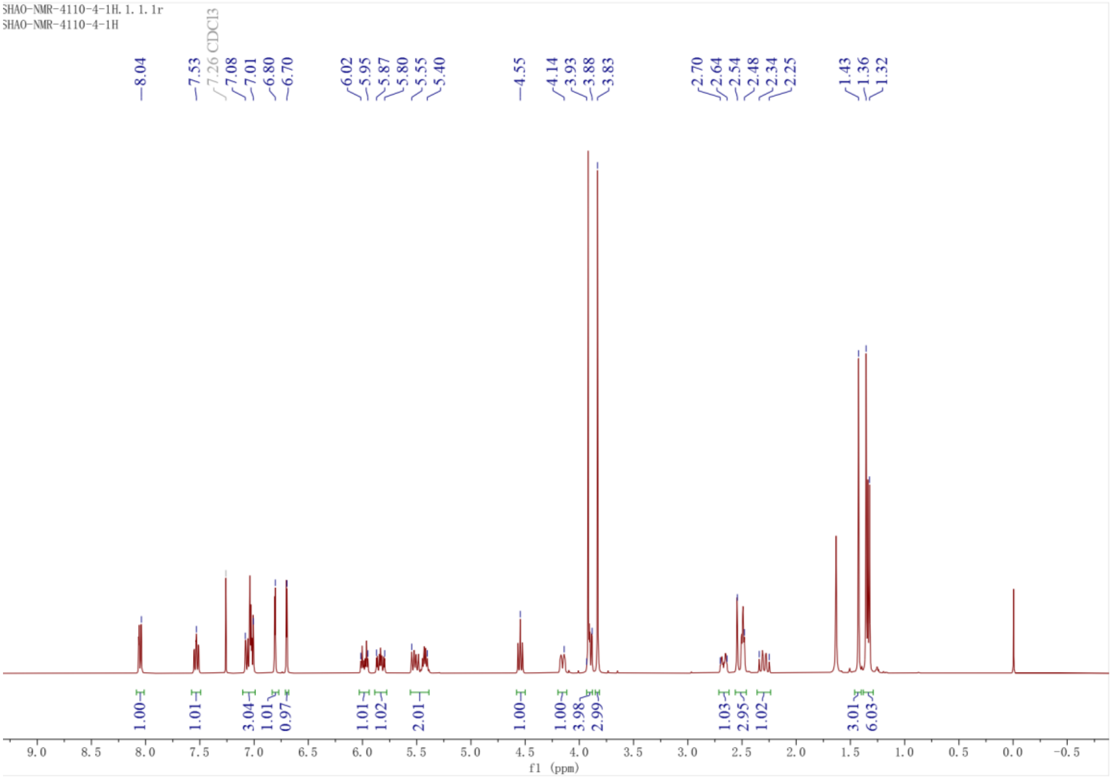

Figure S4. ^{1}H NMR (400 MHz, Chloroform-*d*) spectrum of compound **24**.

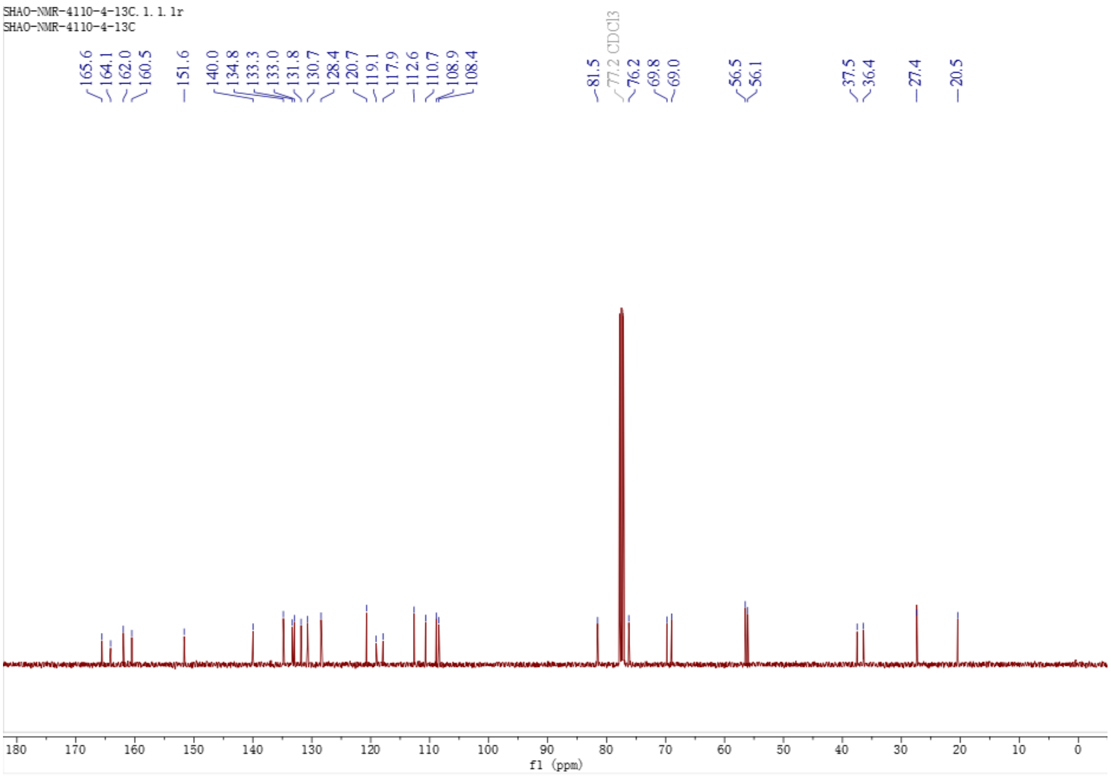

Figure S5. ^{13}C NMR (100 MHz, Chloroform-*d*) spectrum of compound **24**.

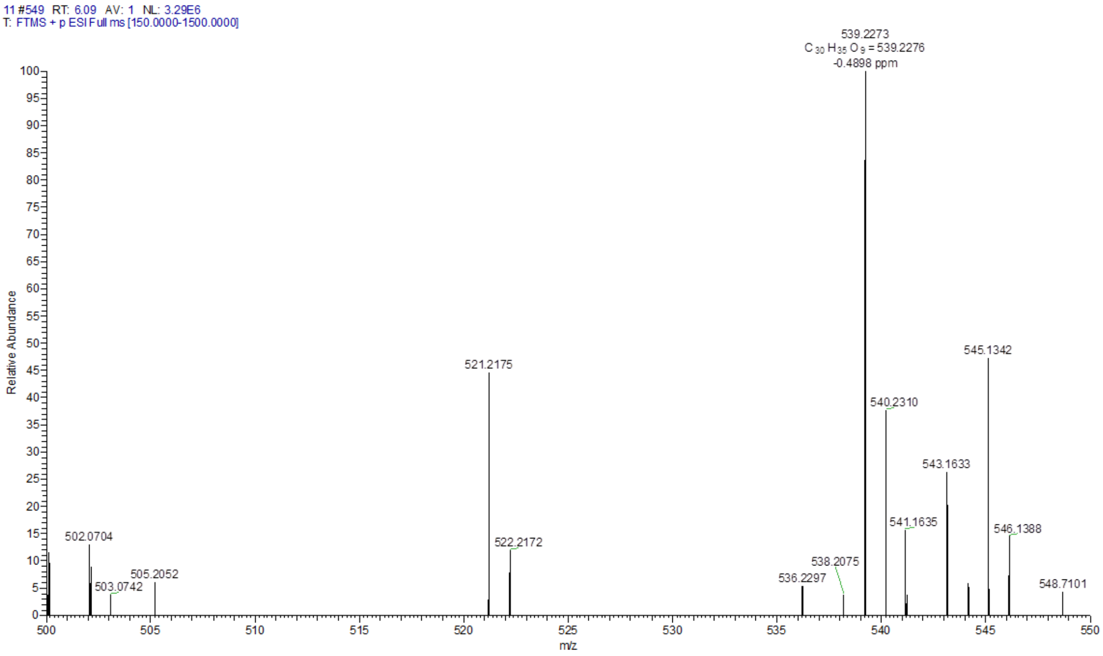

Figure S6. HR-ESI-MS spectrum of compound **24**.

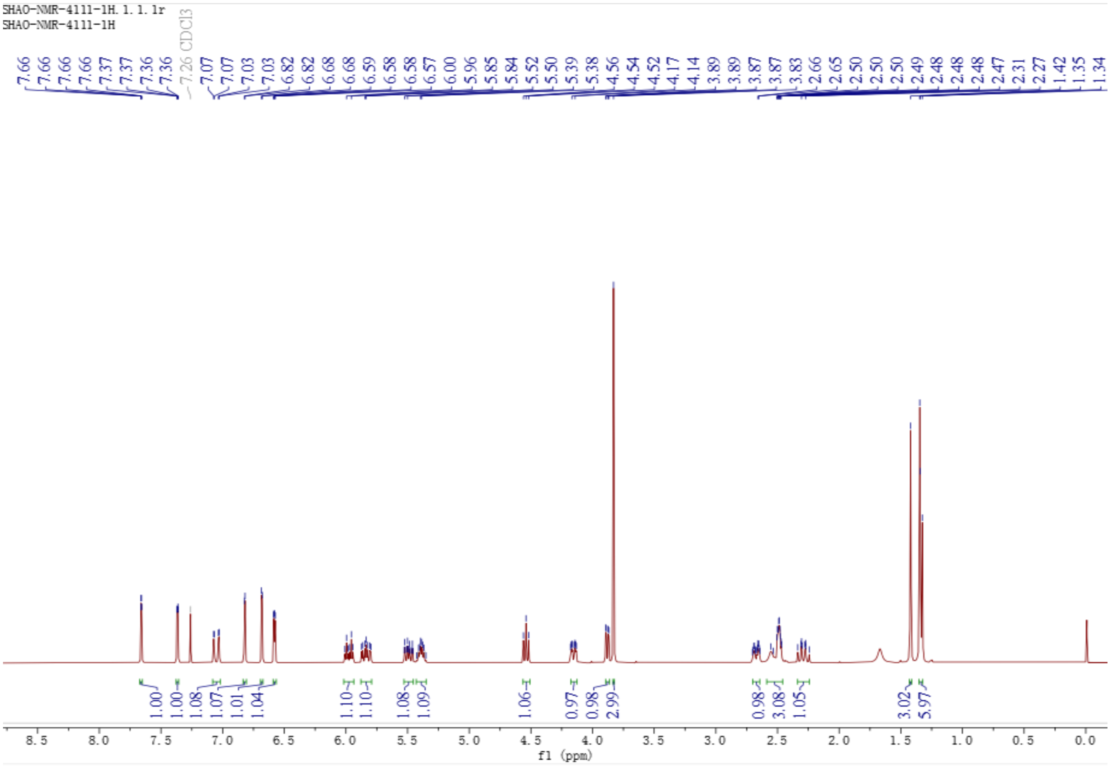

Figure S7. ^{1}H NMR (400 MHz, Chloroform-*d*) spectrum of compound **25**.

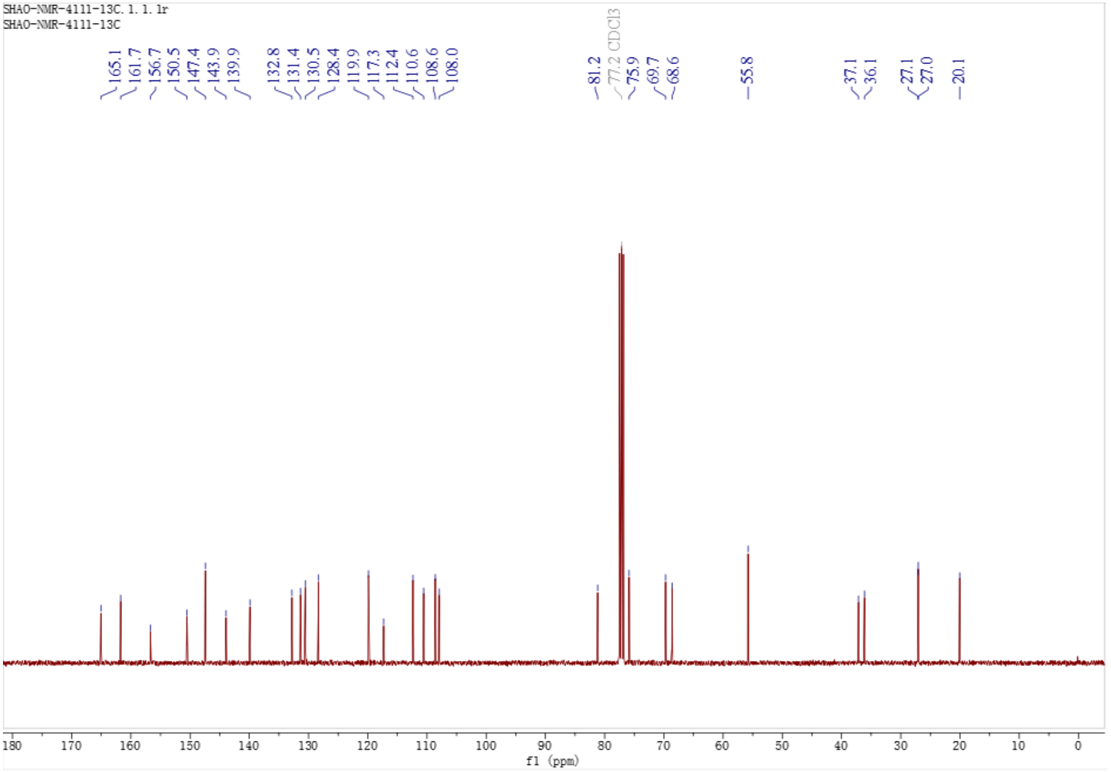

Figure S8. ^{13}C NMR (100 MHz, Chloroform-*d*) spectrum of compound **25**.

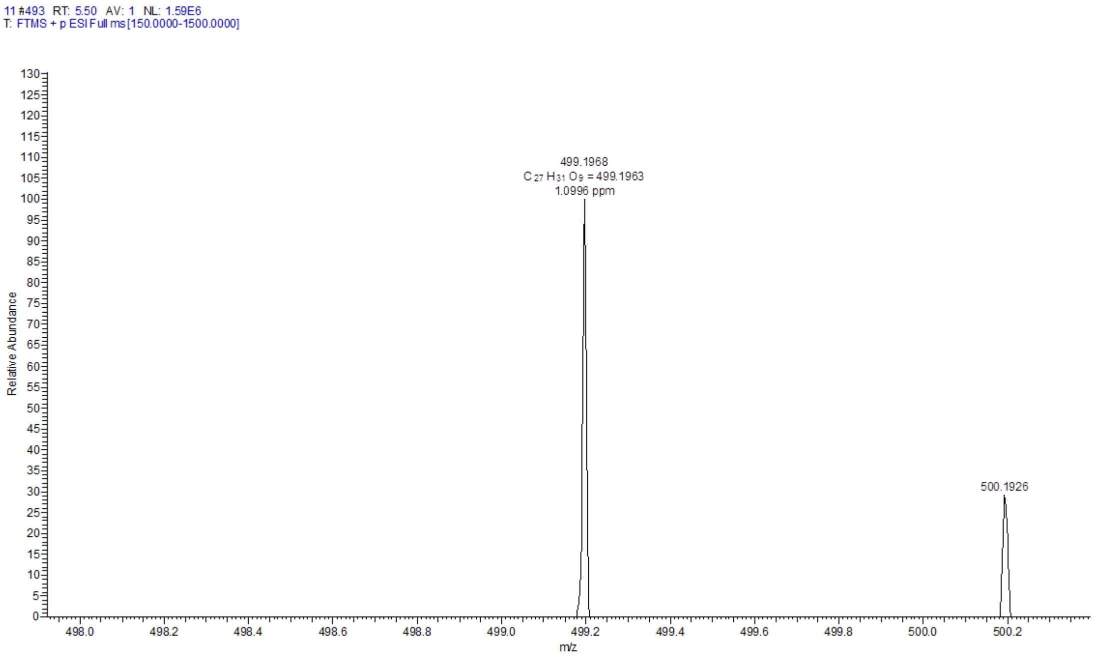

Figure S9. HR-ESI-MS spectrum of compound **25**.

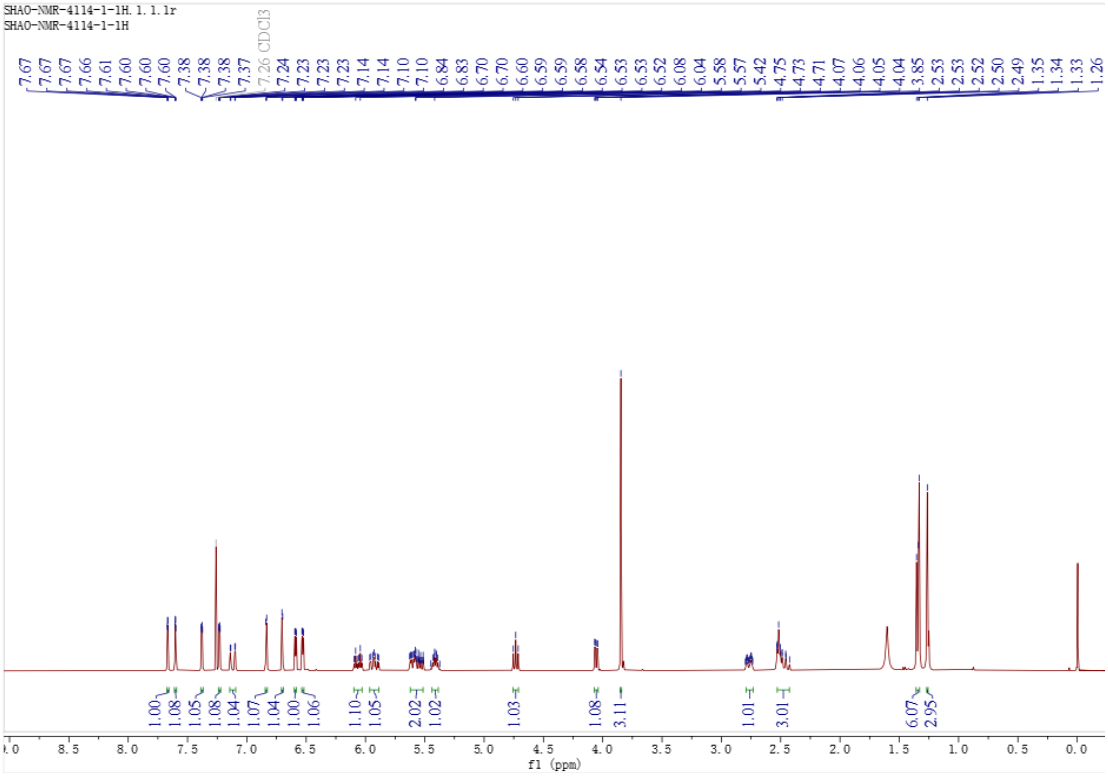

Figure S10. ^{1}H NMR (400 MHz, Chloroform-*d*) spectrum of compound **26**.

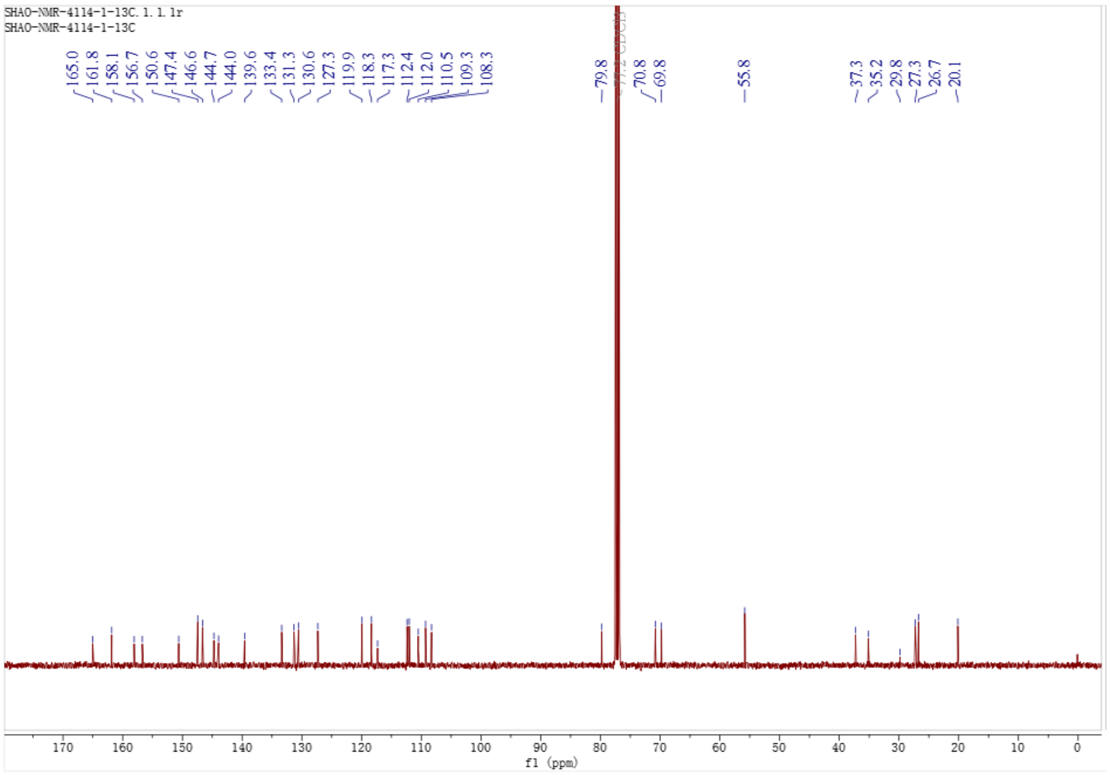

Figure S11. ^{13}C NMR (100 MHz, Chloroform-*d*) spectrum of compound **26**.

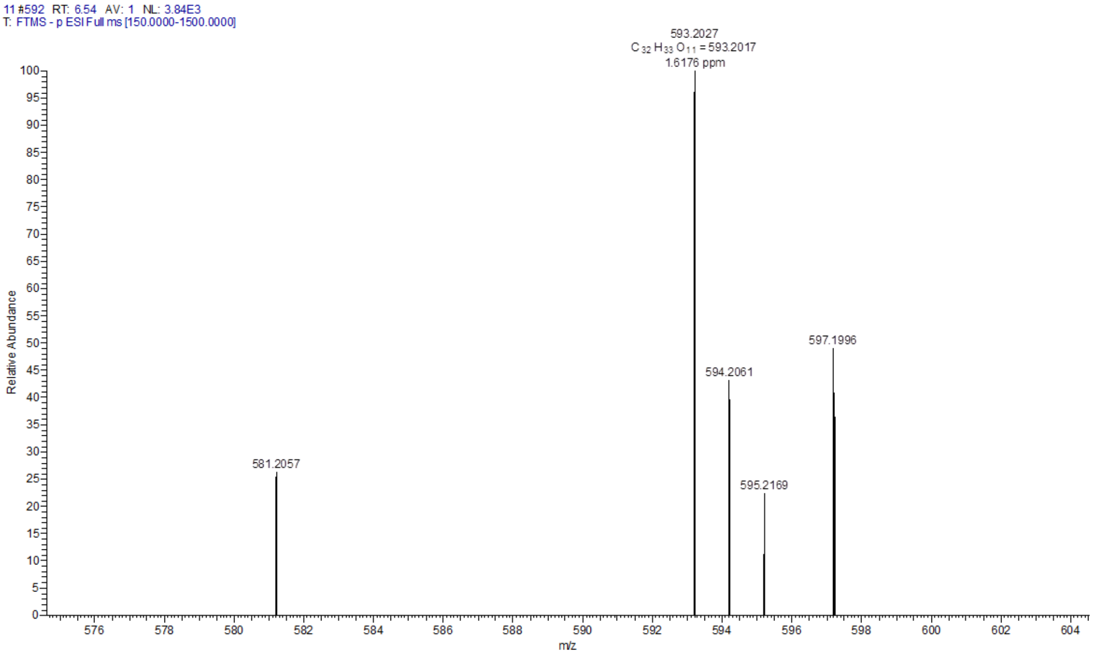

Figure S12. HR-ESI-MS spectrum of compound **26**.

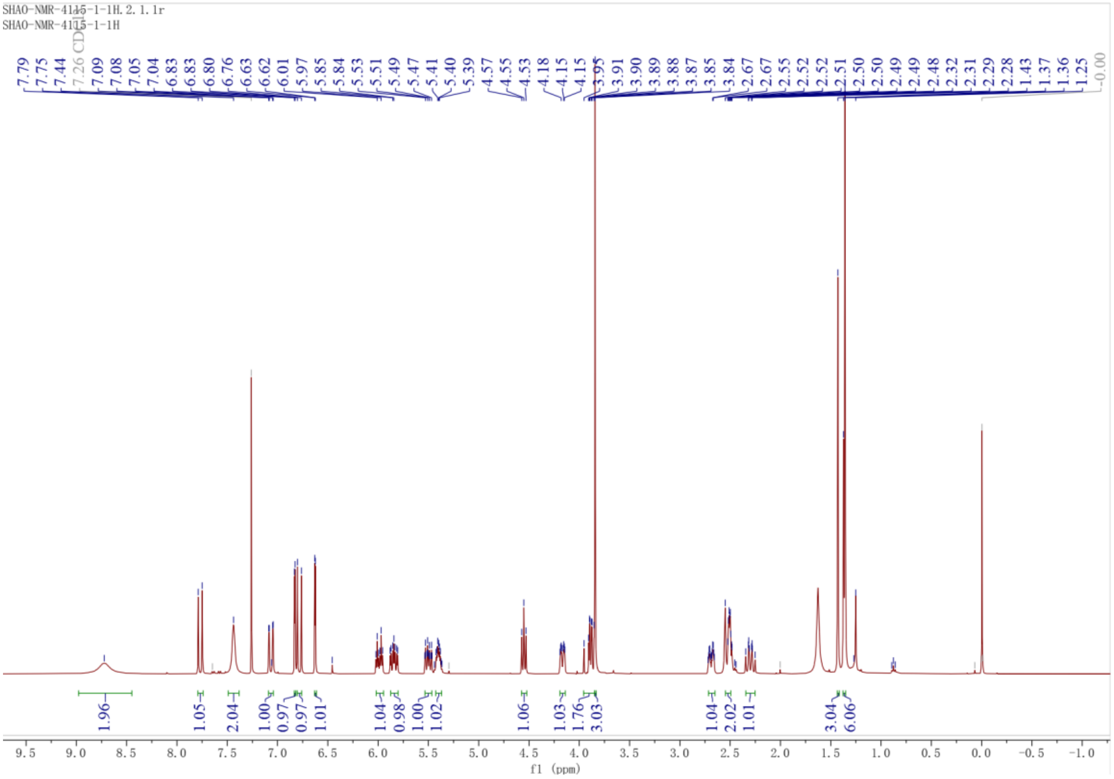

Figure S13. ^{1}H NMR (400 MHz, Chloroform-*d*) spectrum of compound **27**.

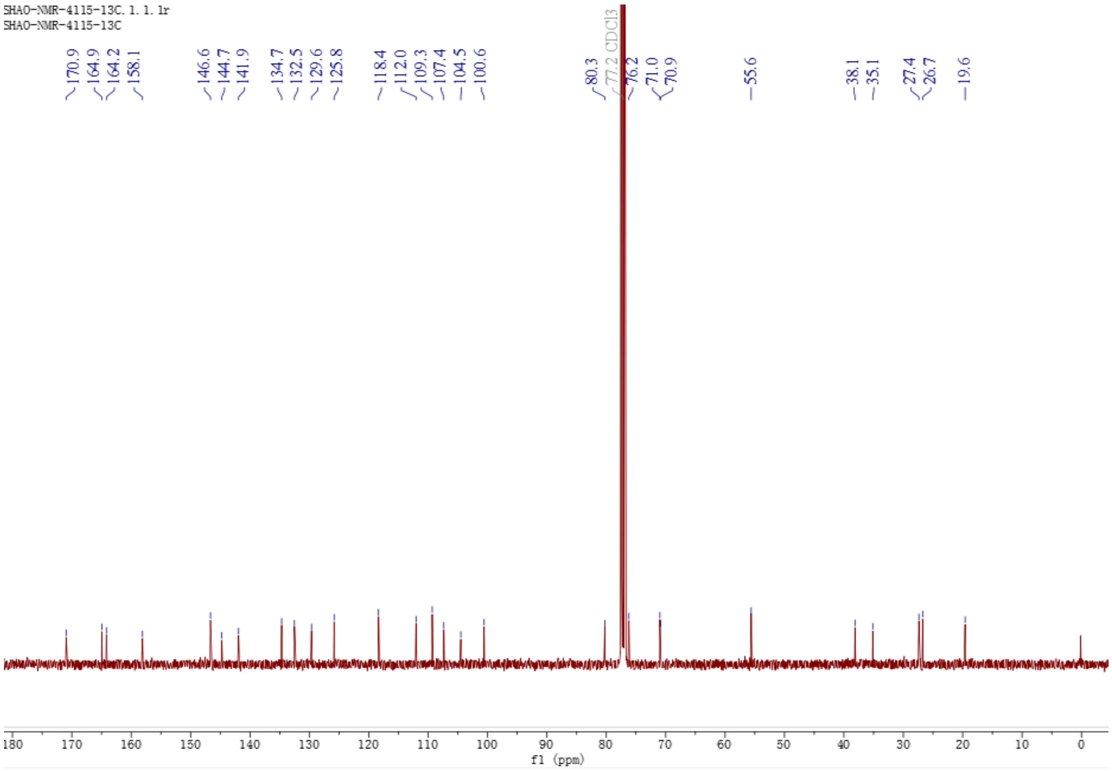

Figure S14. ^{13}C NMR (100 MHz, Chloroform-*d*) spectrum of compound **27**.

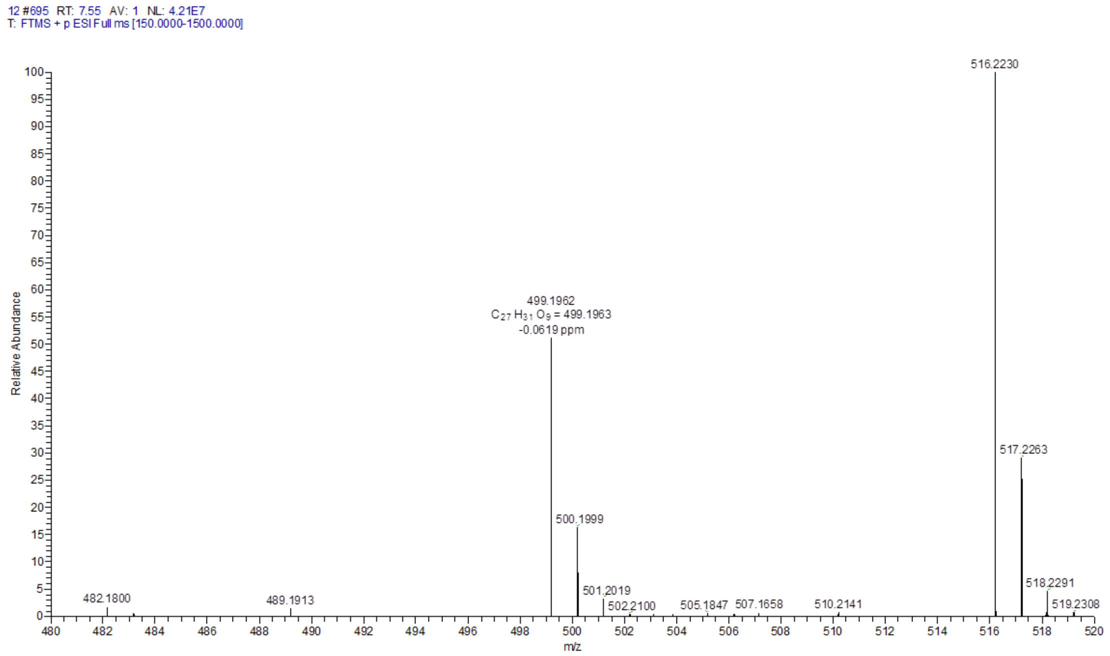

Figure S15. HR-ESI-MS spectrum of compound **27**.

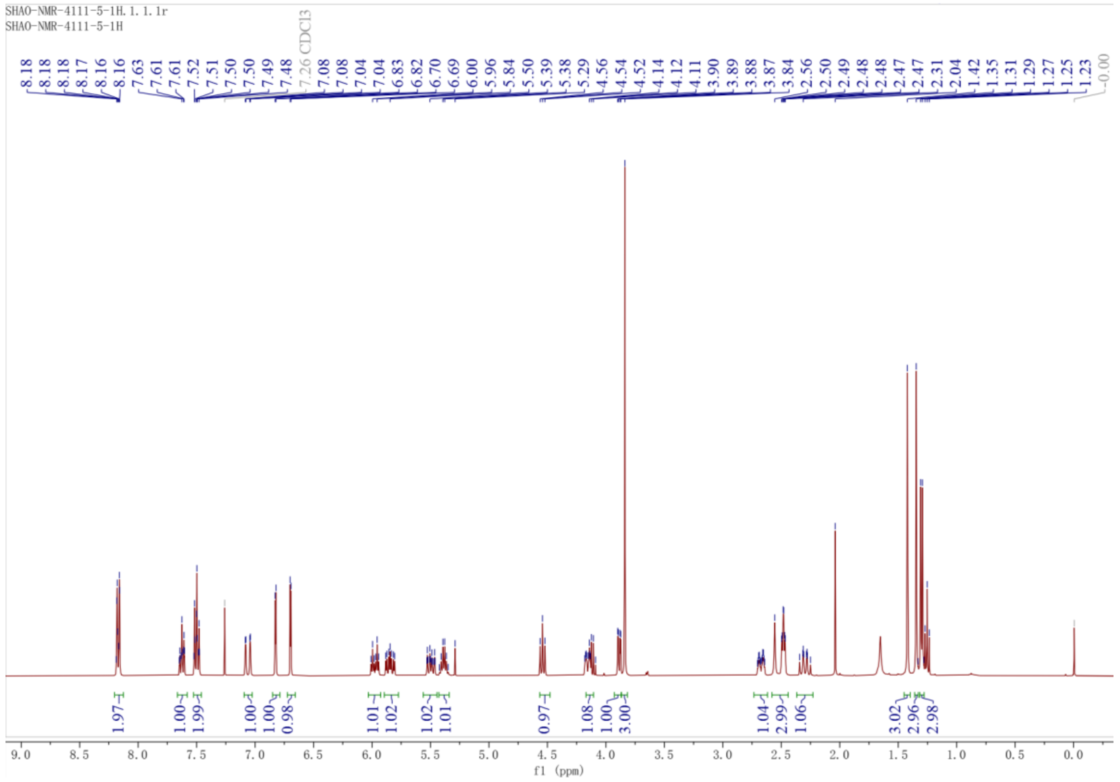

Figure S16. ^{1}H NMR (400 MHz, Chloroform-*d*) spectrum of compound **28**.

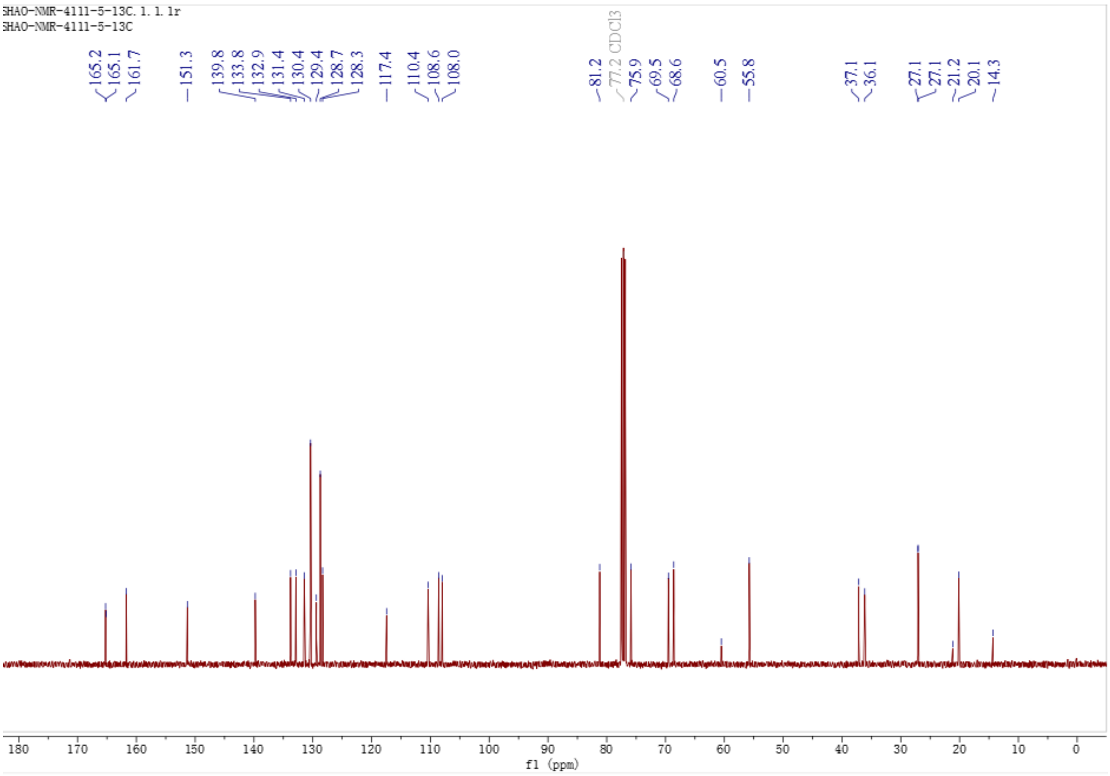

Figure S17. ^{13}C NMR (100 MHz, Chloroform-*d*) spectrum of compound **28**.

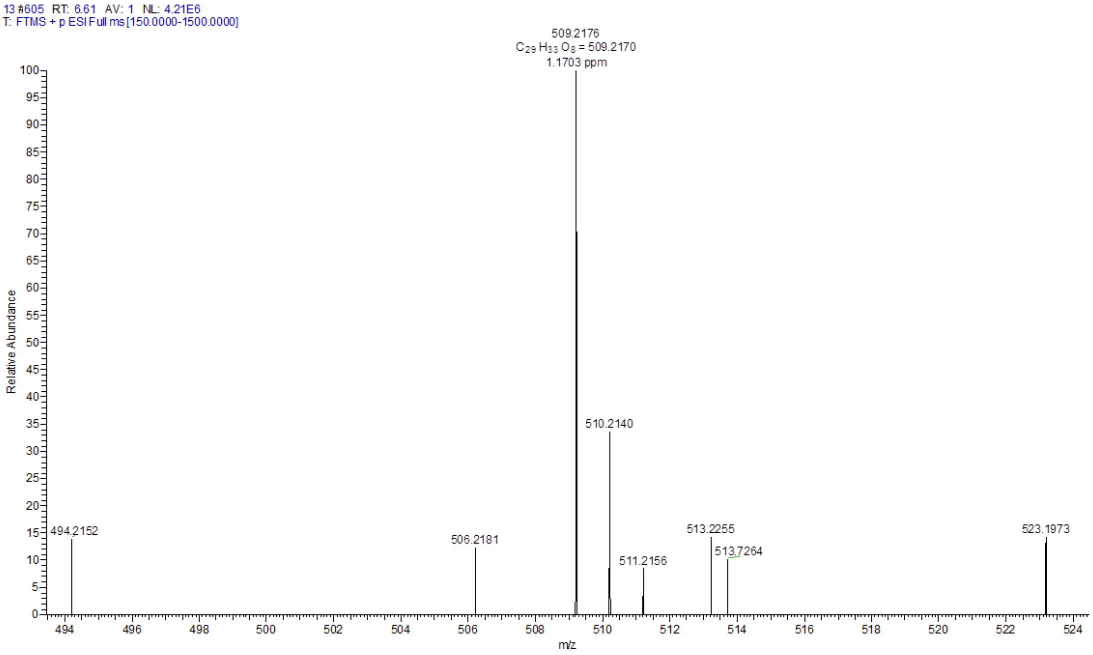

Figure S18. HR-ESI-MS spectrum of compound **28**.

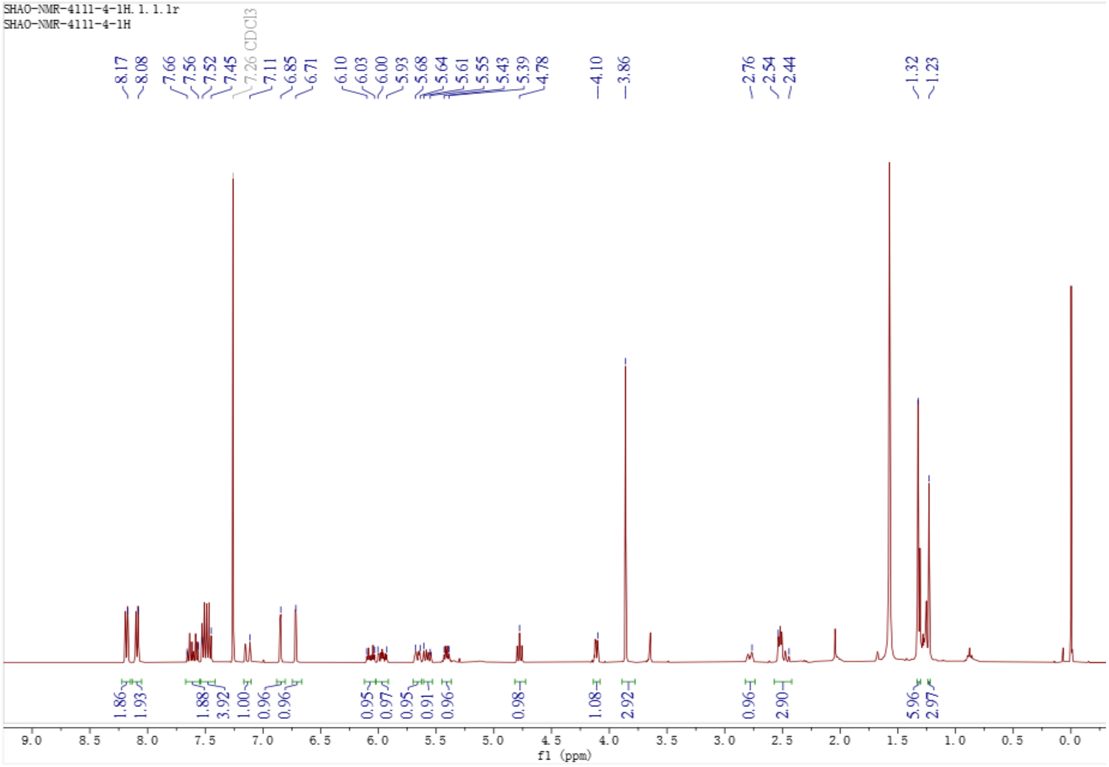

Figure S19. ^{1}H NMR (400 MHz, Chloroform-*d*) spectrum of compound **29**.

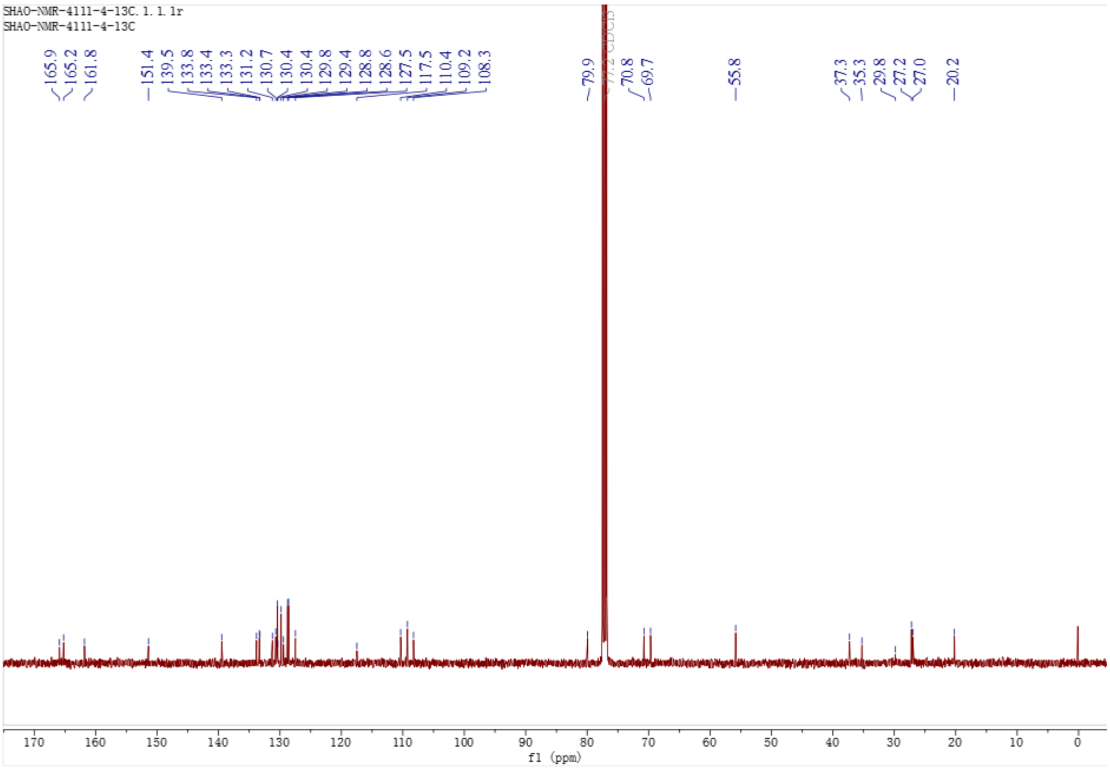

Figure S20. ^{13}C NMR (100 MHz, Chloroform-*d*) spectrum of compound **29**.

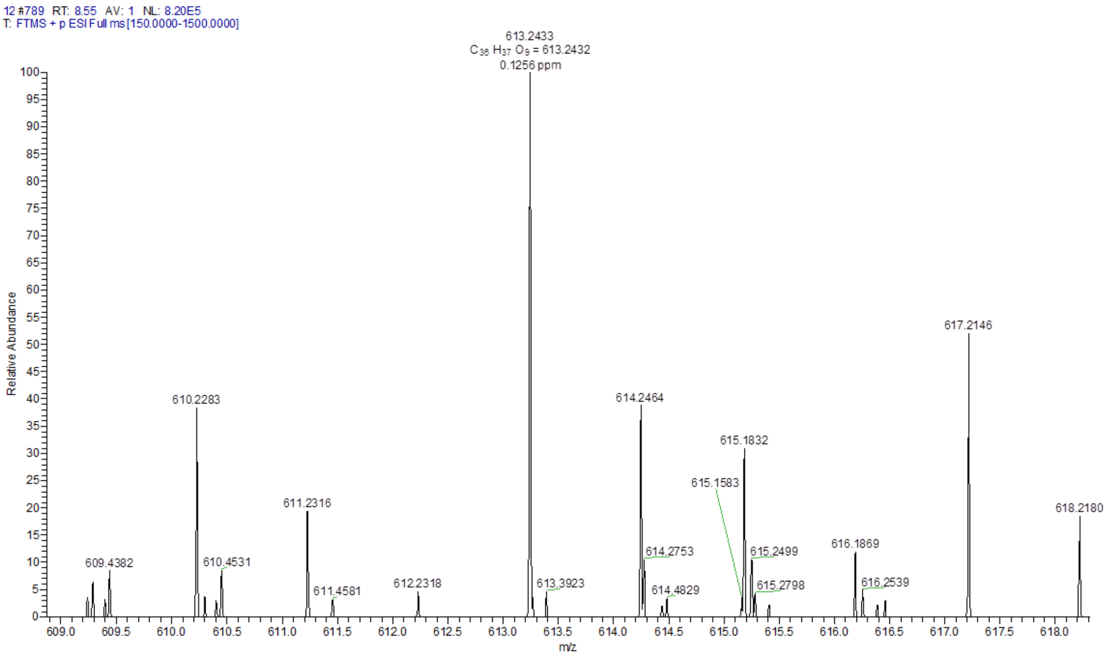

Figure S21. HR-ESI-MS spectrum of compound **29**.

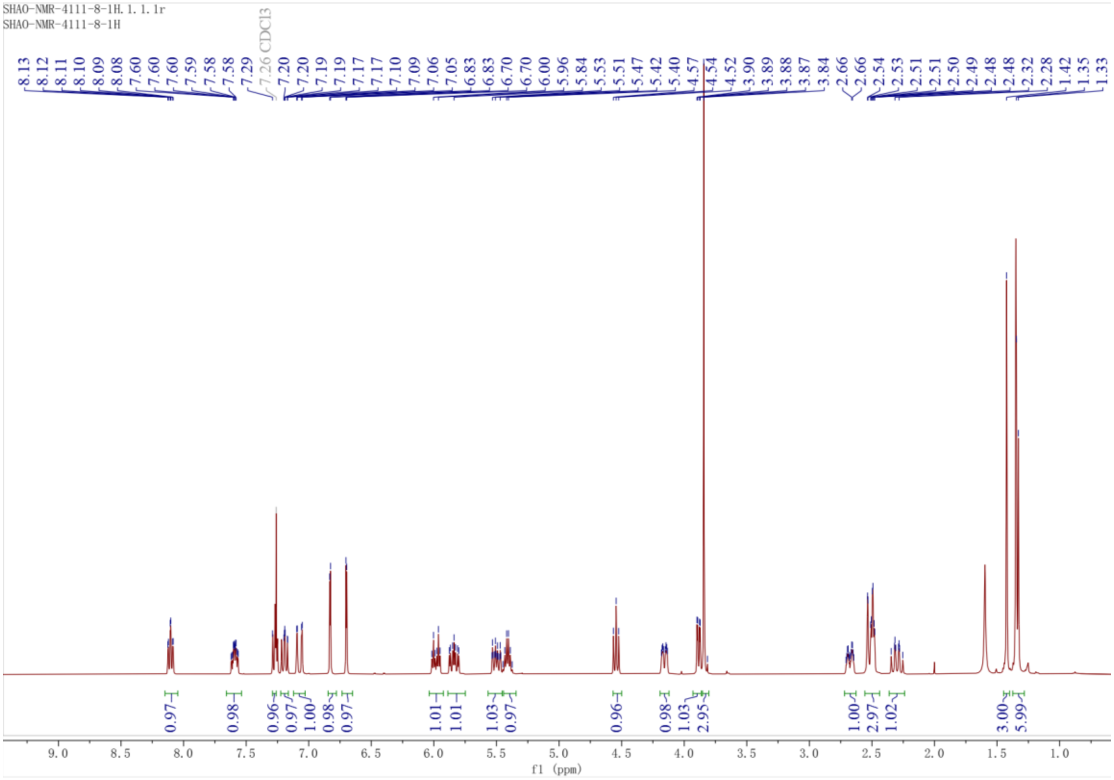

Figure S22. ^{1}H NMR (400 MHz, Chloroform-*d*) spectrum of compound **30**.

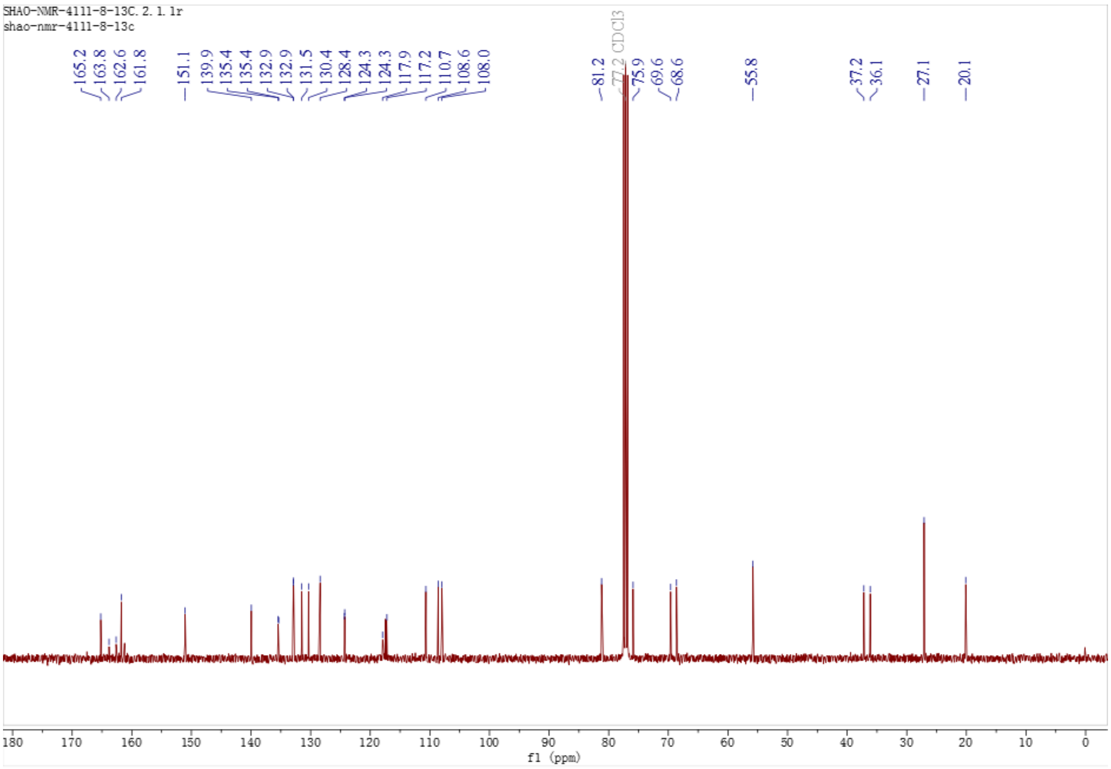

Figure S23. ^{13}C NMR (100 MHz, Chloroform-*d*) spectrum of compound **30**.

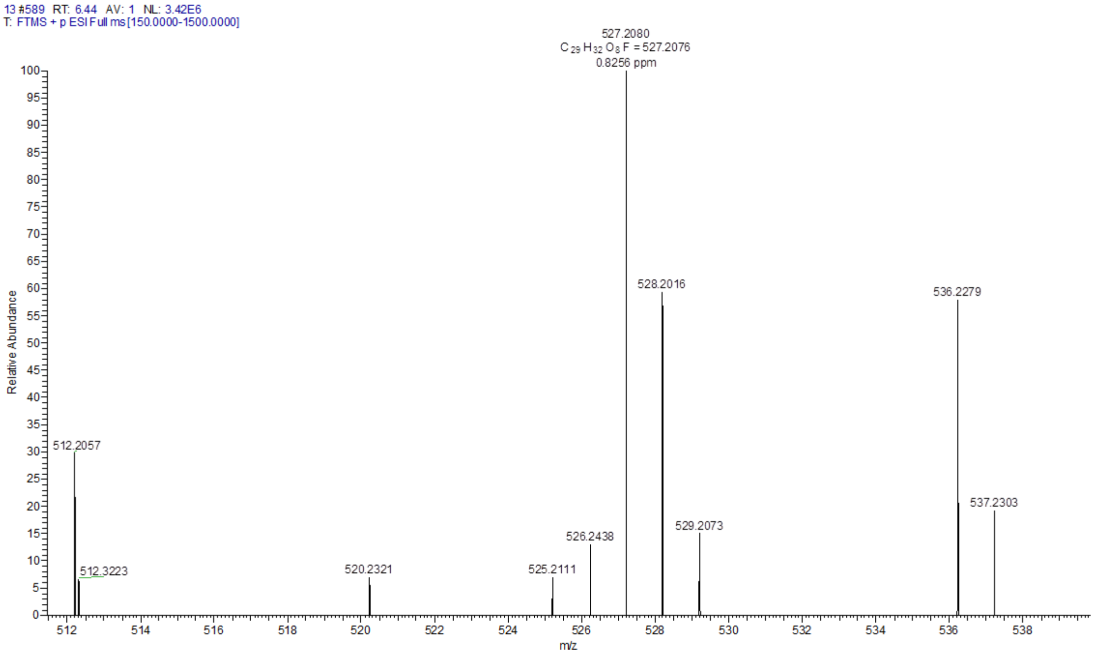

Figure S24. HR-ESI-MS spectrum of compound **30**.

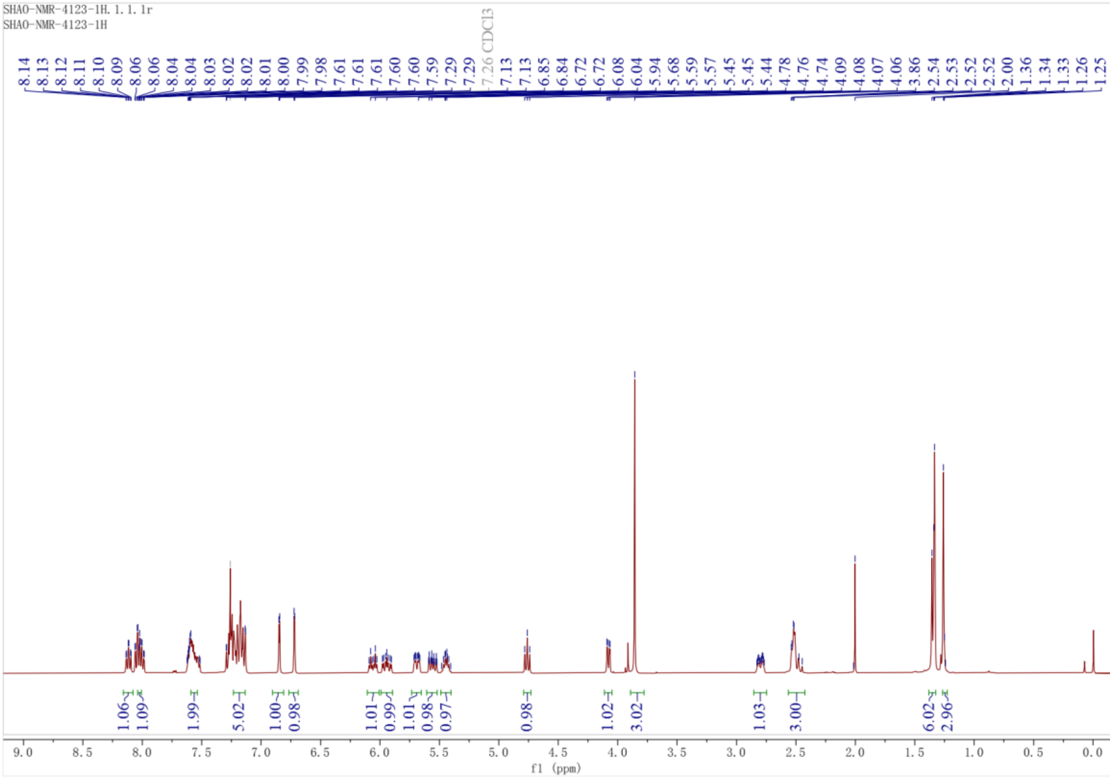

Figure S25. ^{1}H NMR (400 MHz, Chloroform-*d*) spectrum of compound **31**.

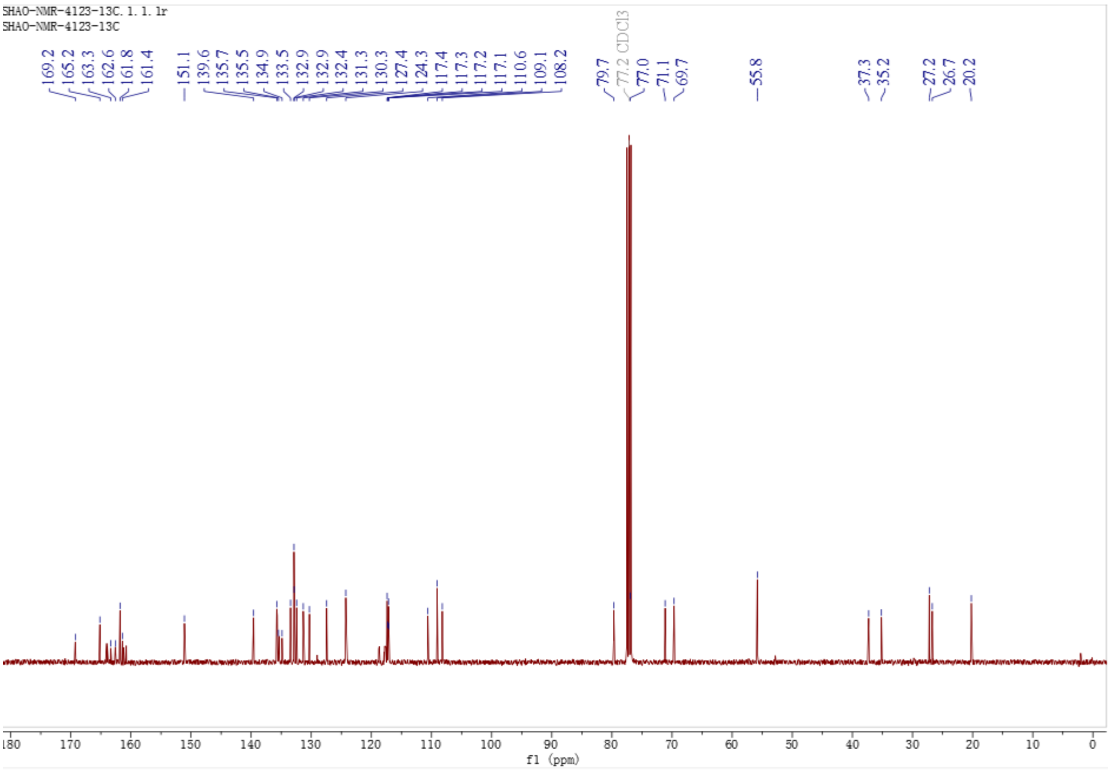

Figure S26. ^{13}C NMR (100 MHz, Chloroform-*d*) spectrum of compound **31**.

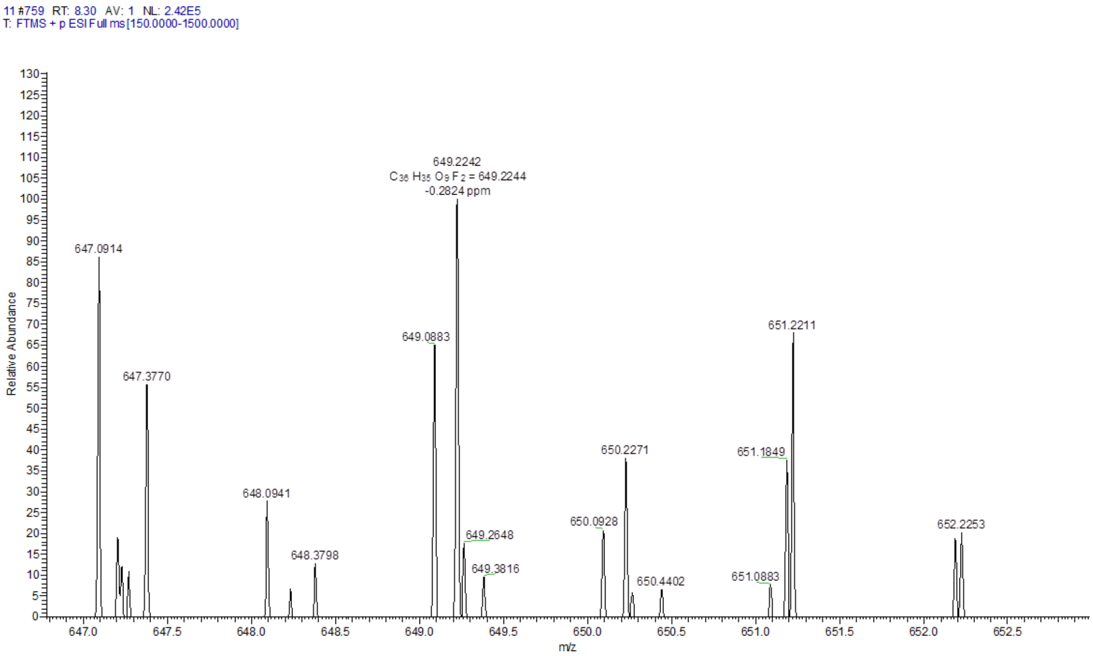

Figure S27. HR-ESI-MS spectrum of compound **31**.

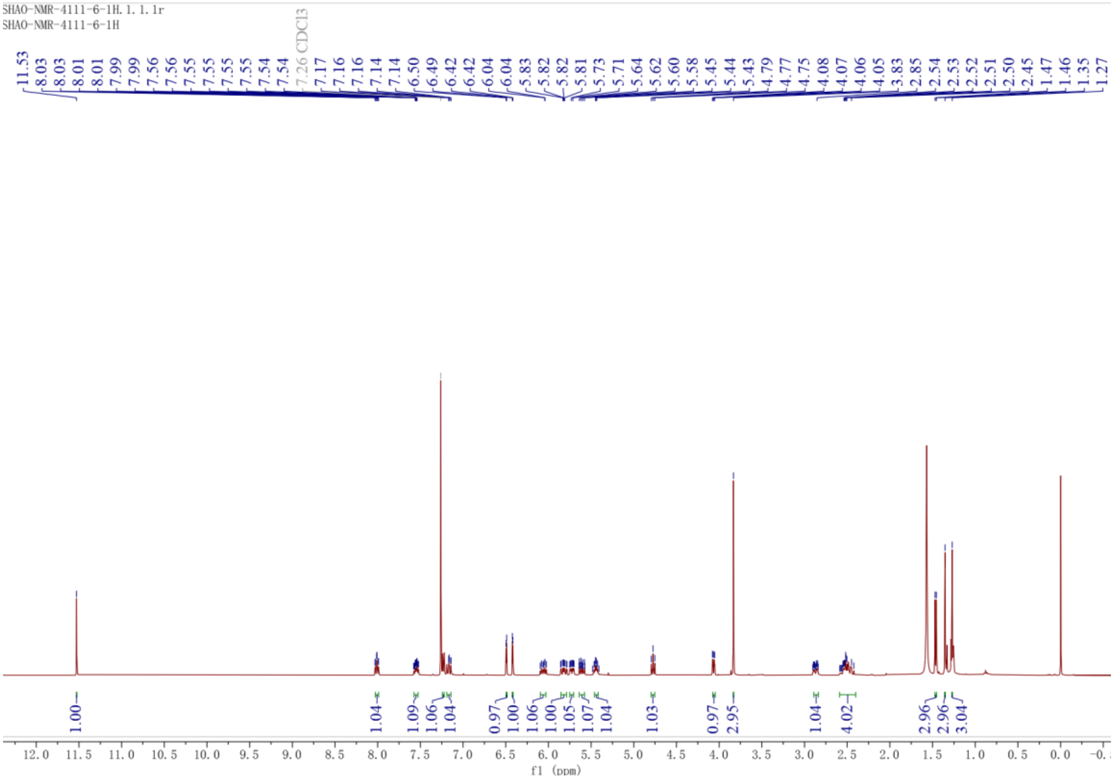

Figure S28. ^{1}H NMR (400 MHz, Chloroform-*d*) spectrum of compound **32**.

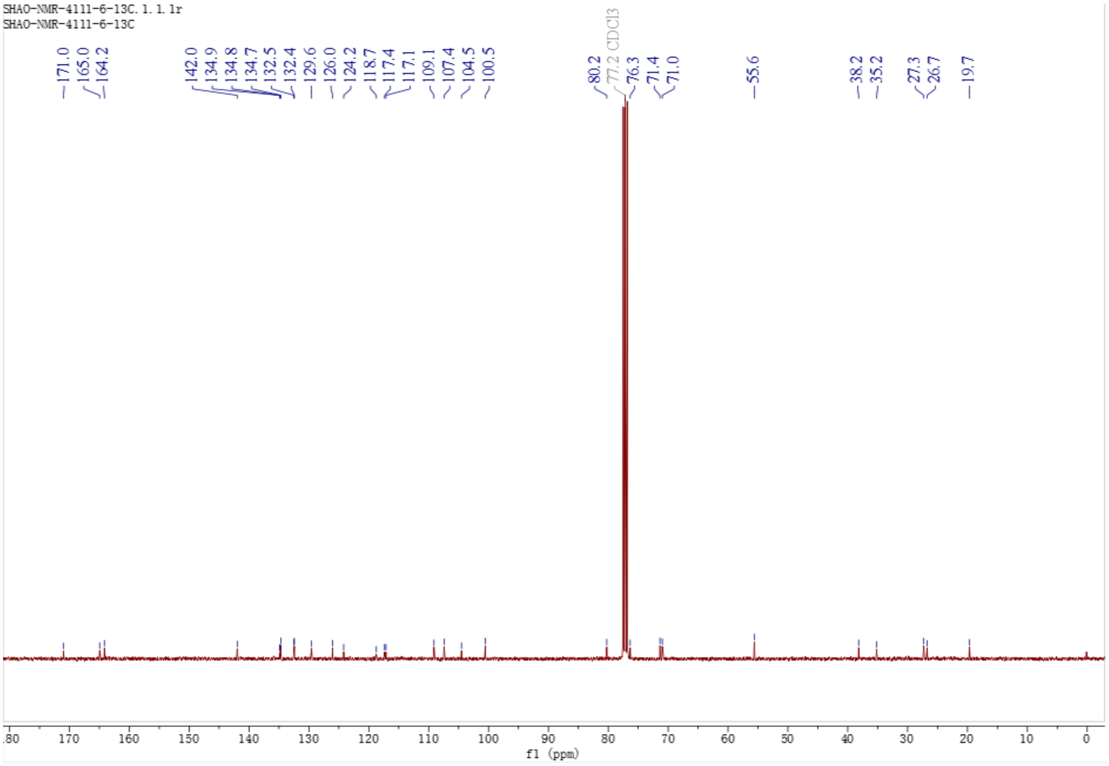

Figure S29. ^{13}C NMR (100 MHz, Chloroform-*d*) spectrum of compound **32**.

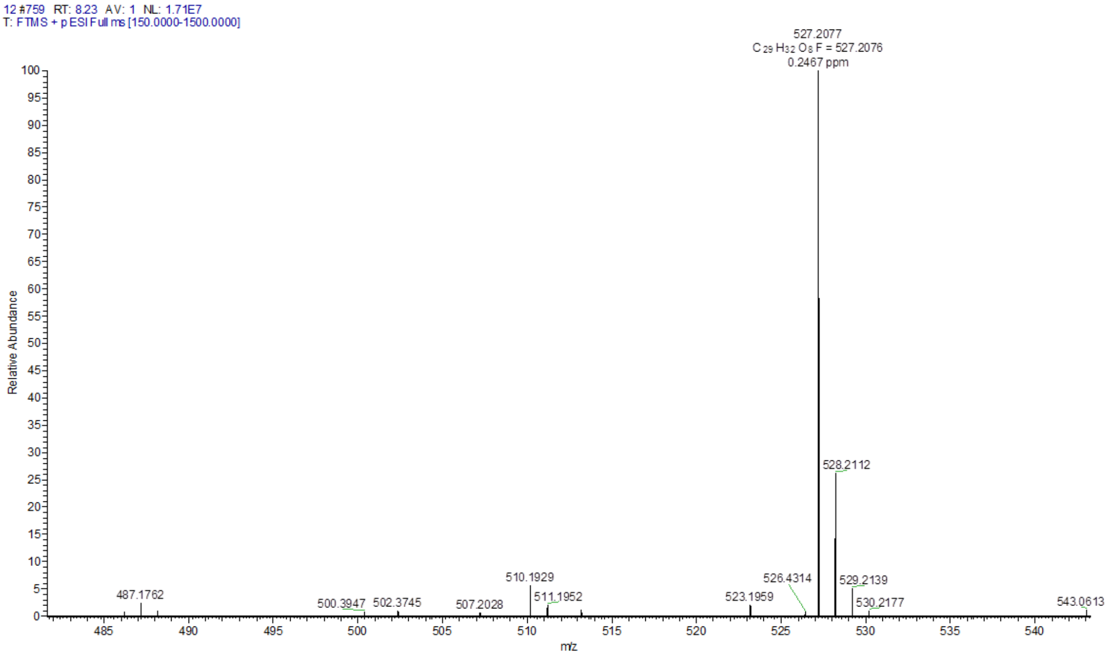

Figure S30. HR-ESI-MS spectrum of compound **32**.

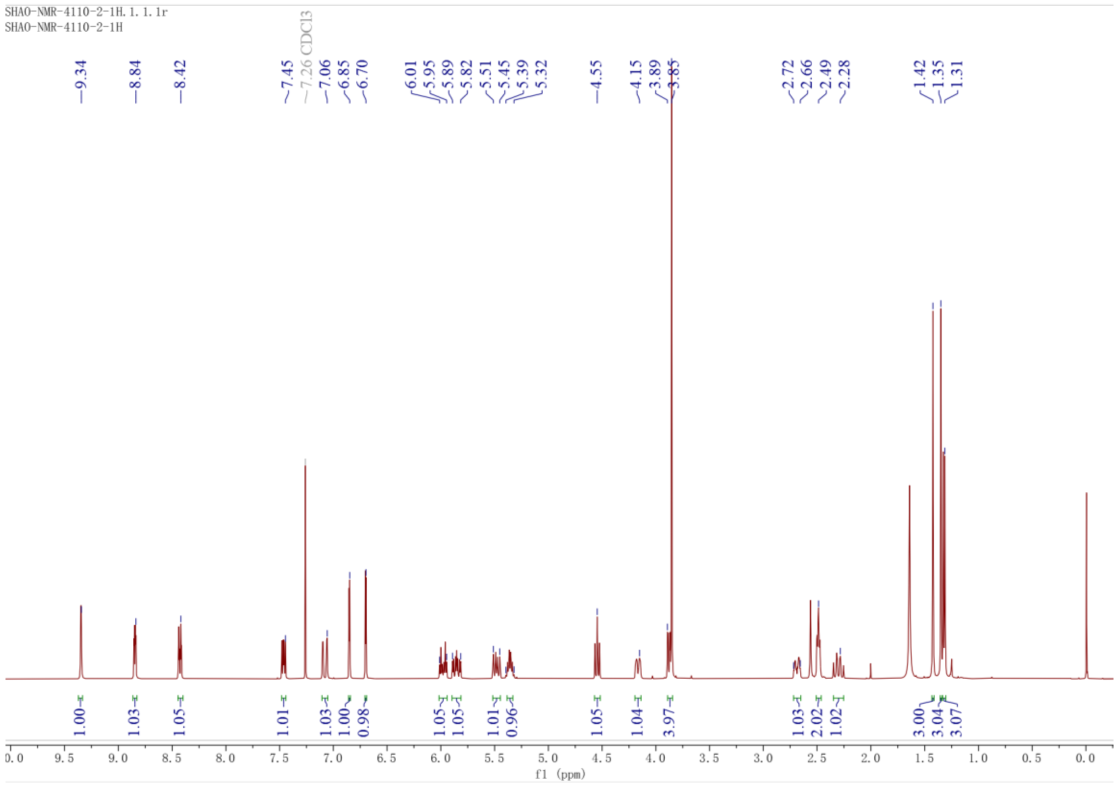

Figure S31. ^{1}H NMR (400 MHz, Chloroform-*d*) spectrum of compound **33**.

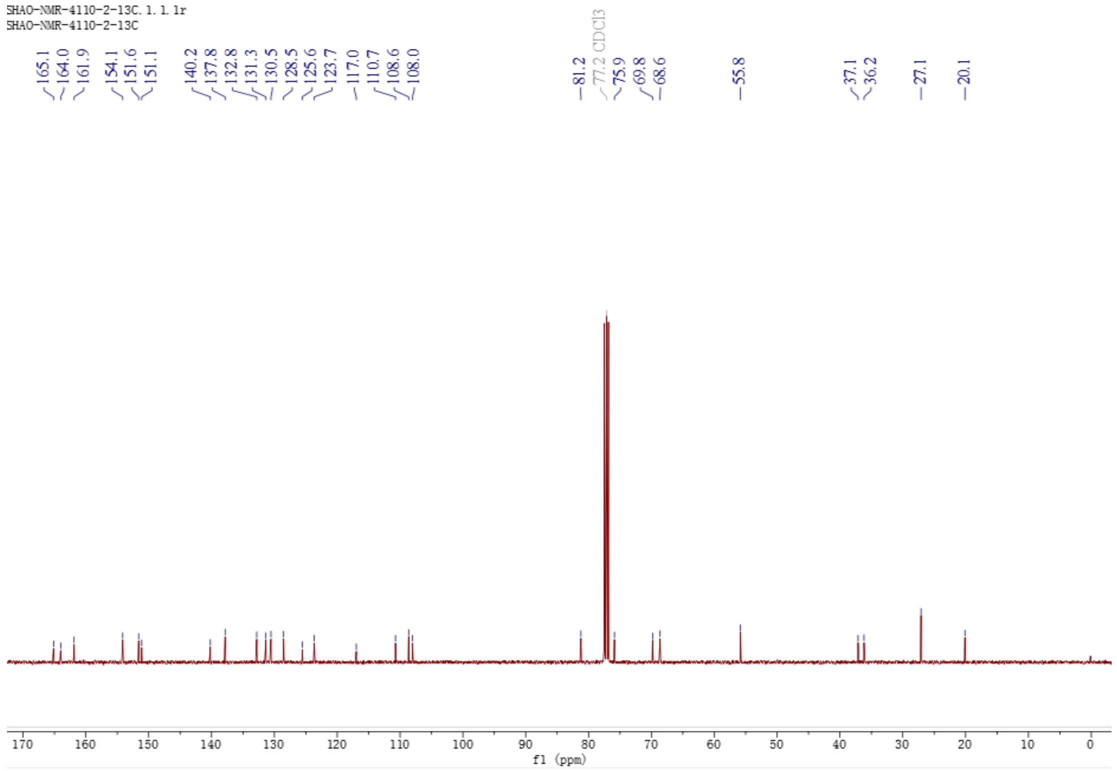

Figure S32. ^{13}C NMR (100 MHz, Chloroform-*d*) spectrum of compound **33**.

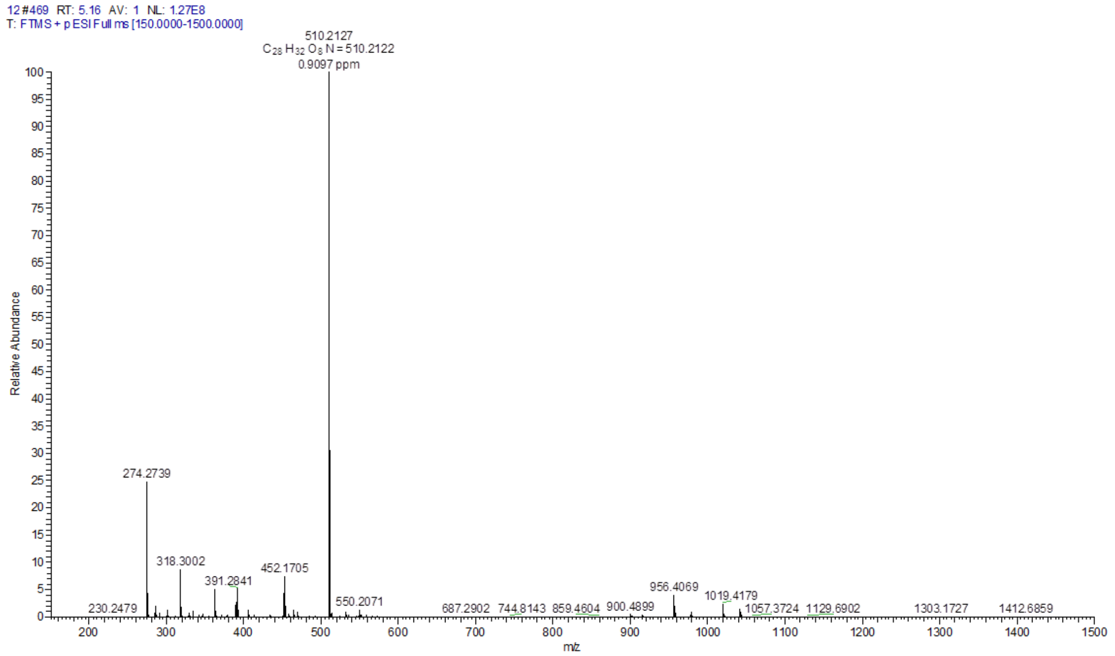

Figure S33. HR-ESI-MS spectrum of compound **33**.

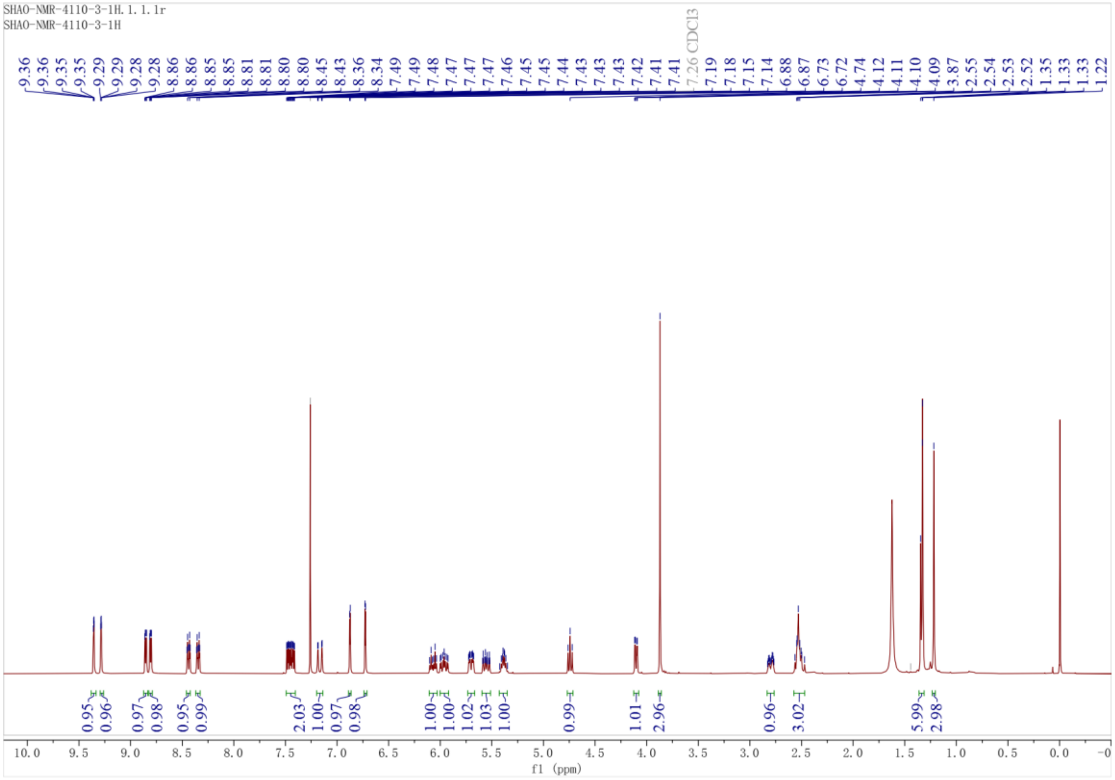

Figure S34. ^{1}H NMR (400 MHz, Chloroform-*d*) spectrum of compound **34**.

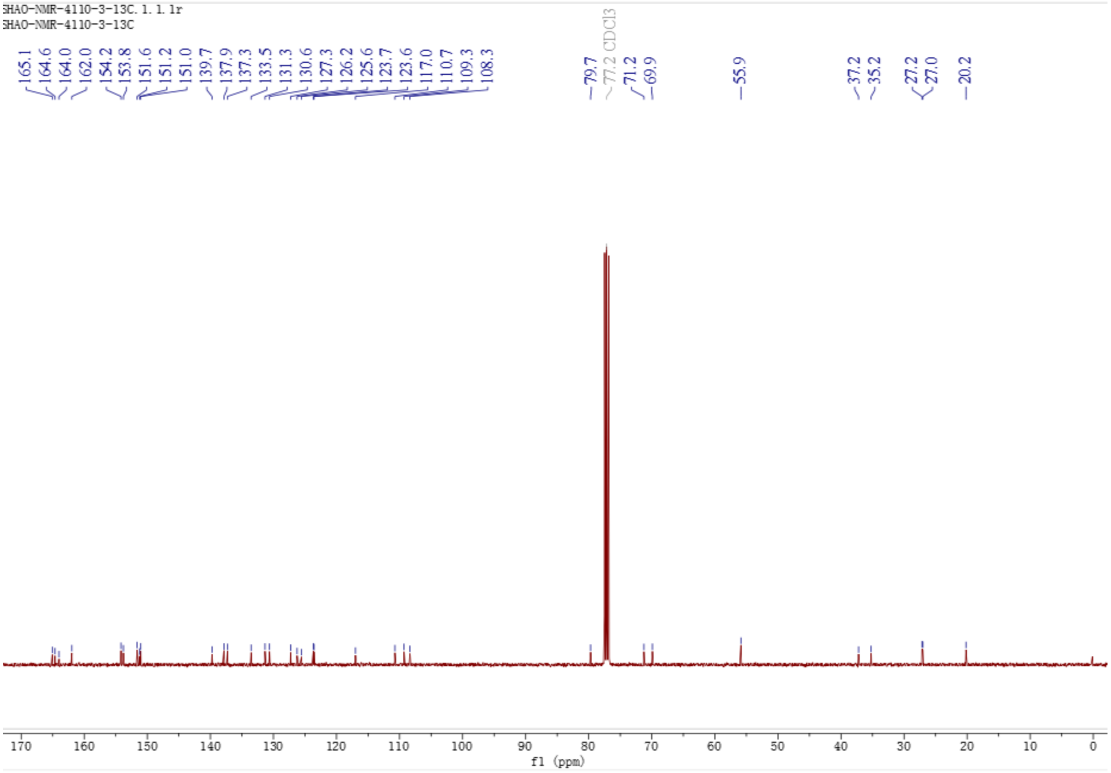

Figure S35 ^{13}C NMR (100 MHz, Chloroform-*d*) spectrum of compound **34**.

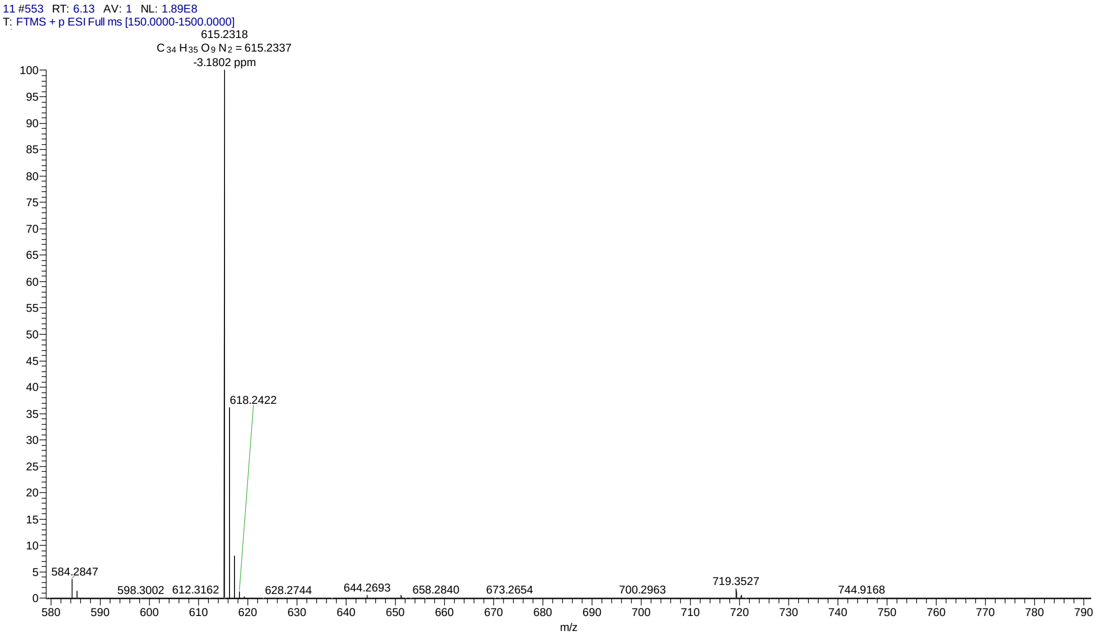

Figure S36. HR-ESI-MS spectrum of compound **34**.

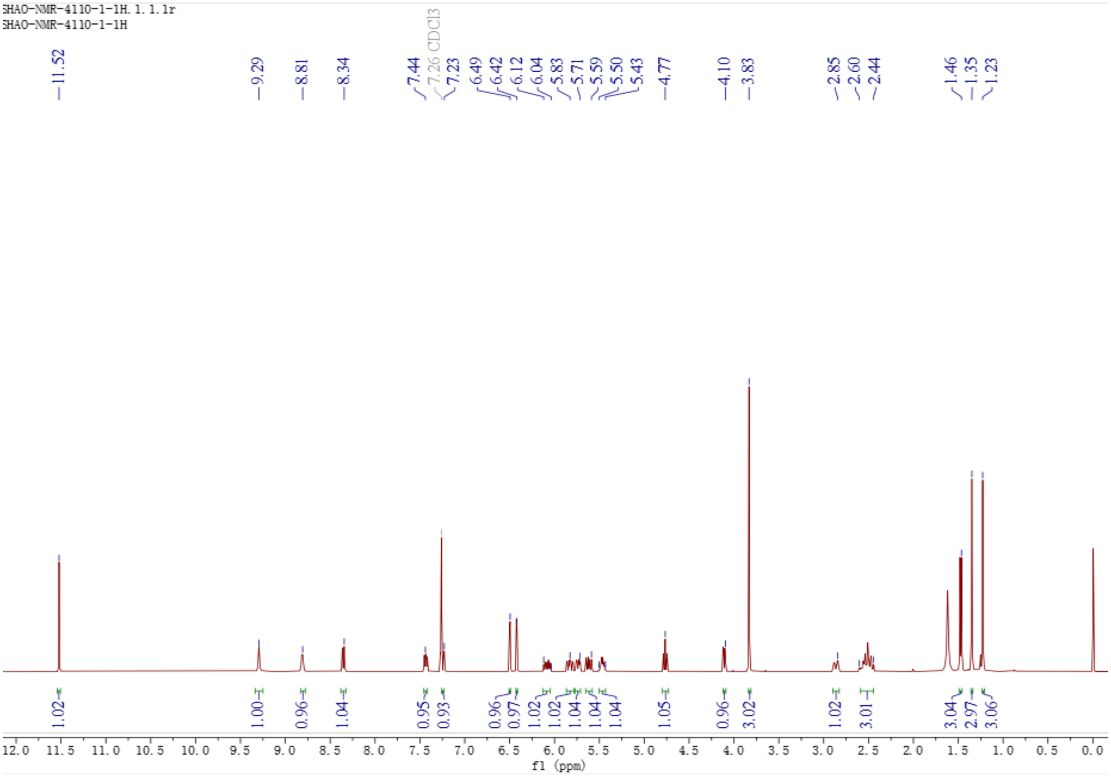

Figure S37. ^{1}H NMR (400 MHz, Chloroform-*d*) spectrum of compound **35**.

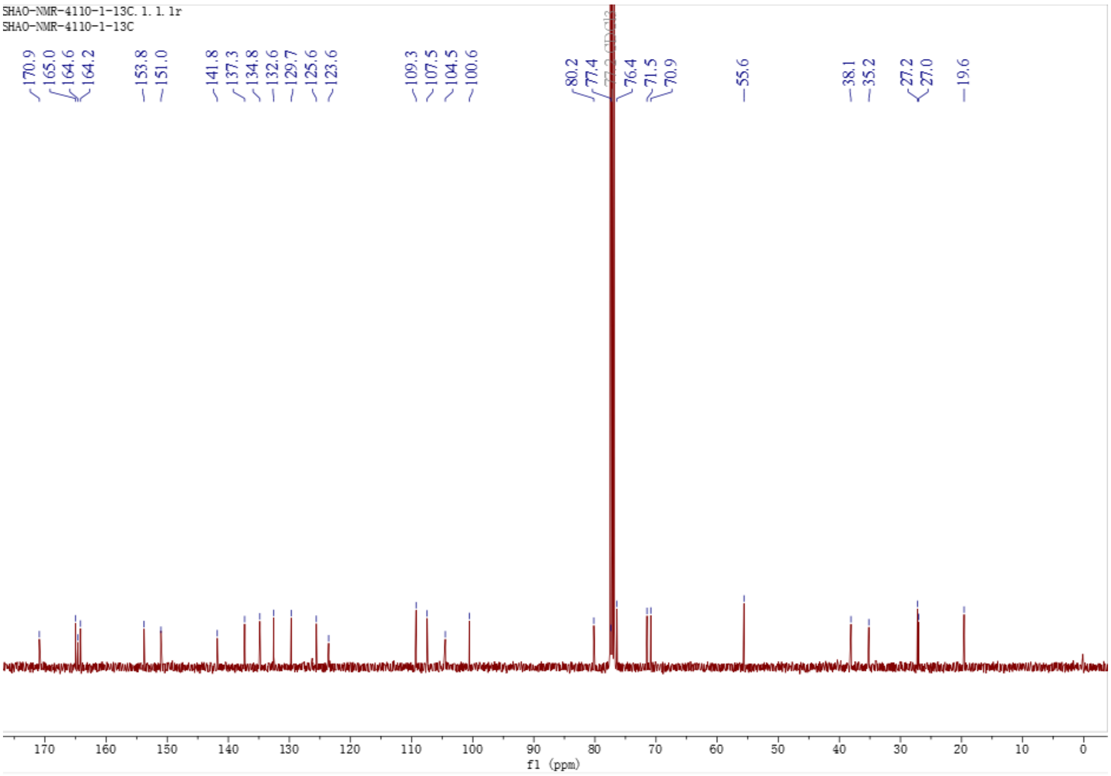

Figure S38. ^{13}C NMR (100 MHz, Chloroform-*d*) spectrum of compound **35**.

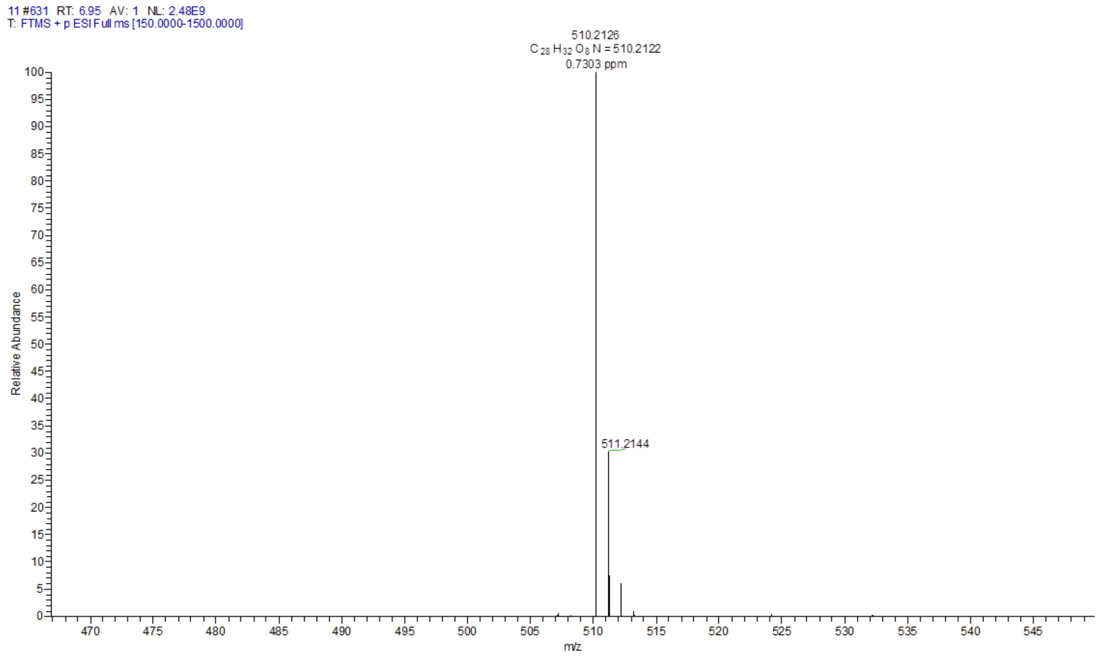

Figure S39. HR-ESI-MS spectrum of compound **35**.

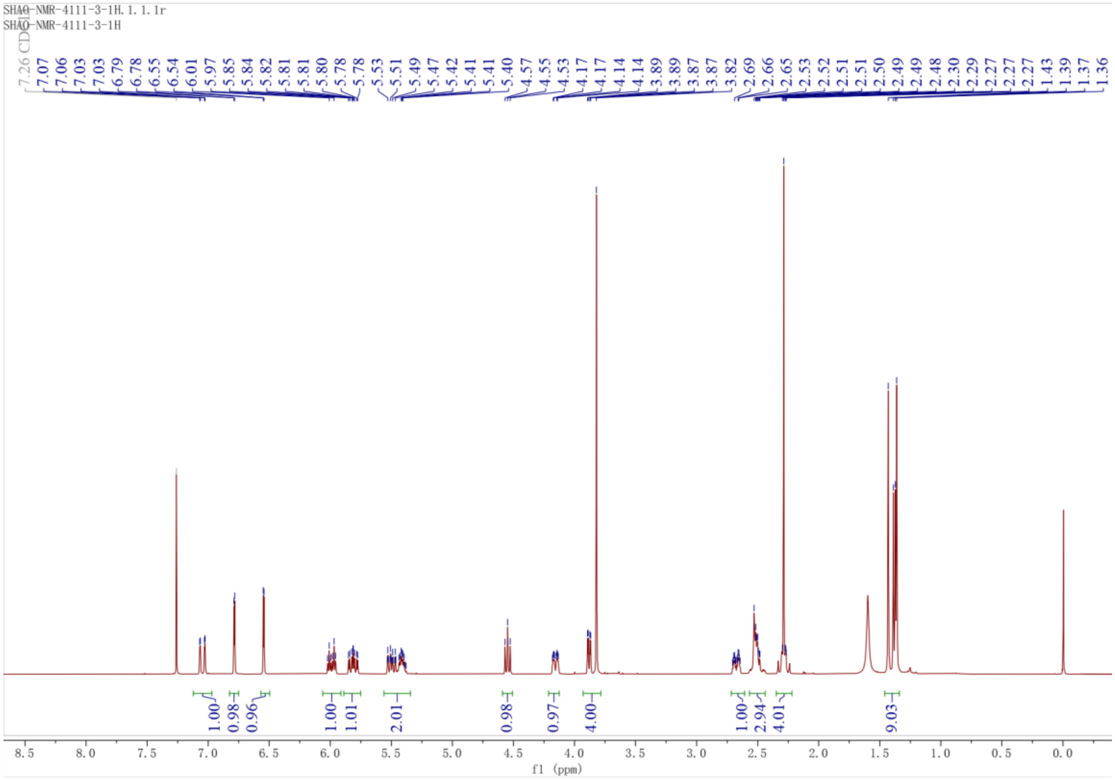

Figure S40. ^{1}H NMR (400 MHz, Chloroform-*d*) spectrum of compound **36**.

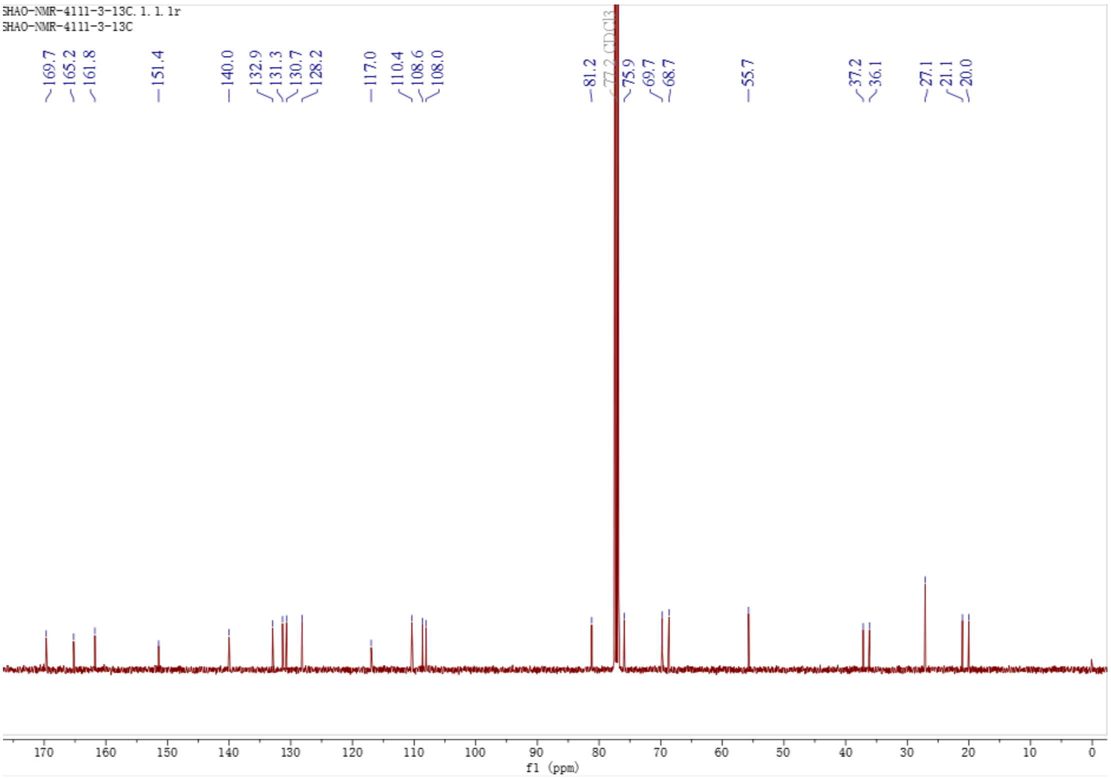

Figure S41. ^{13}C NMR (100 MHz, Chloroform-*d*) spectrum of compound **36**.

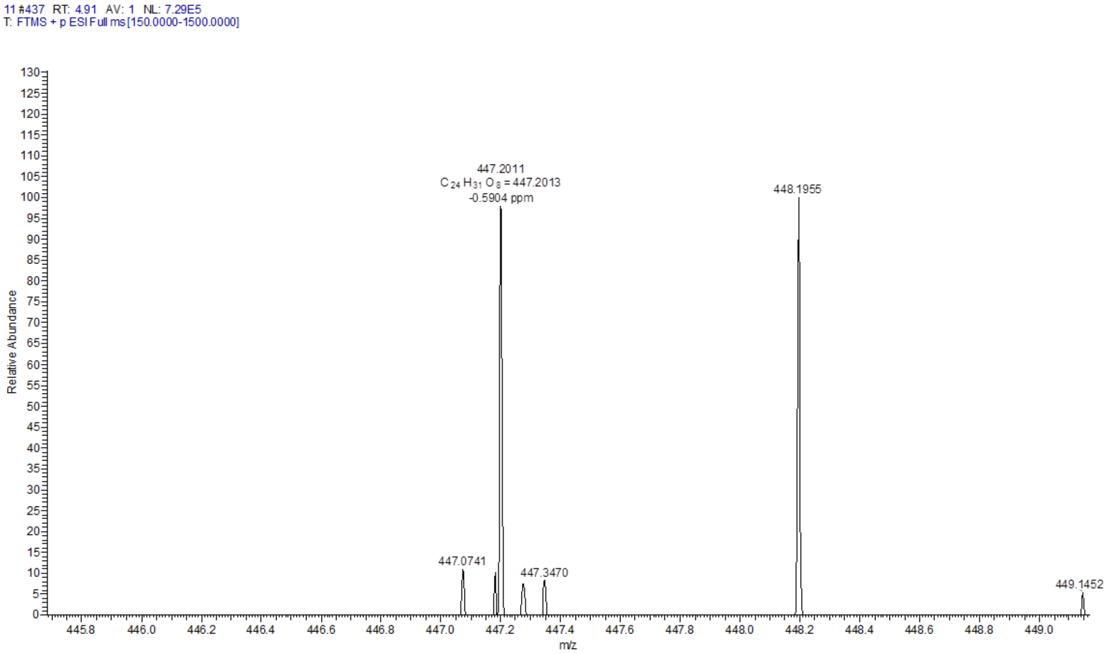

Figure S42. HR-ESI-MS spectrum of compound **36**

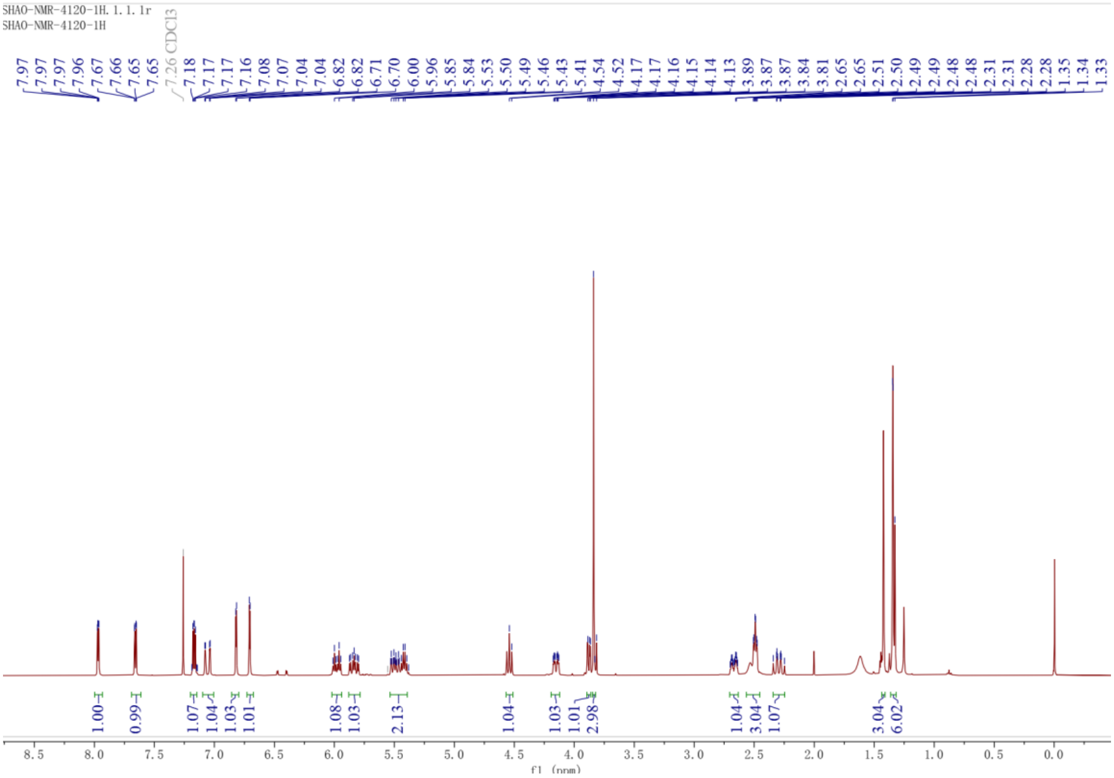

Figure S43. ^{1}H NMR (400 MHz, Chloroform-*d*) spectrum of compound **37**.

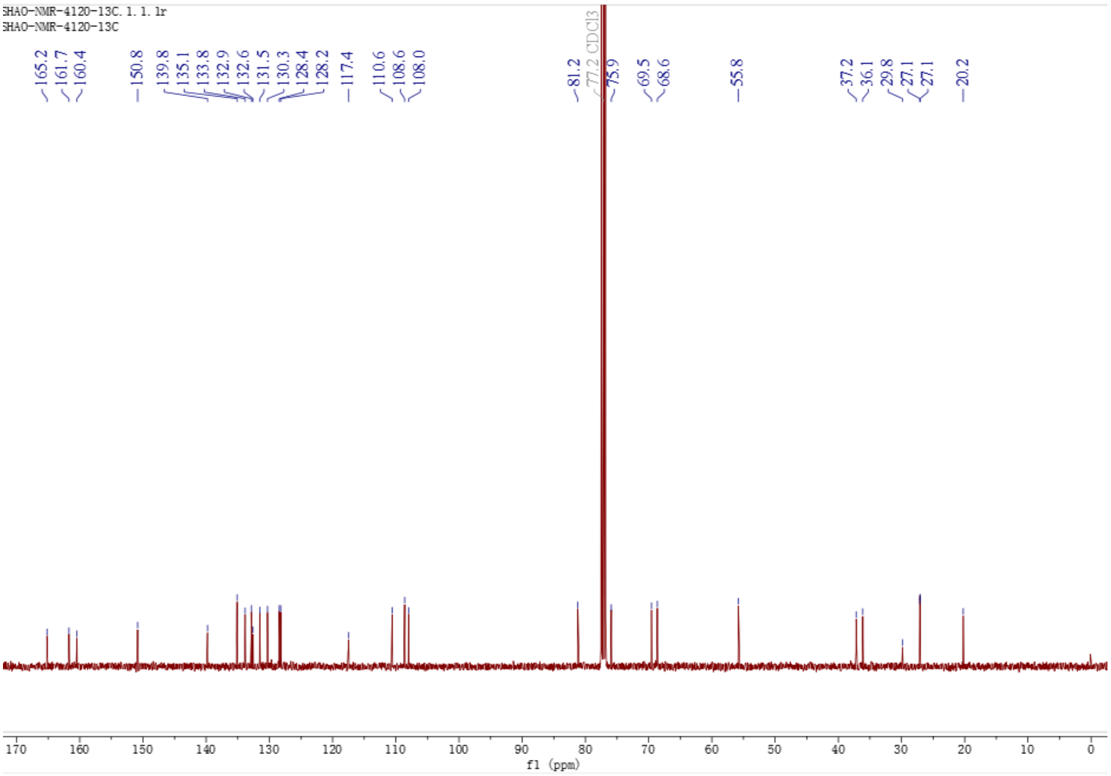

Figure S44. ^{13}C NMR (100 MHz, Chloroform-*d*) spectrum of compound **37**.

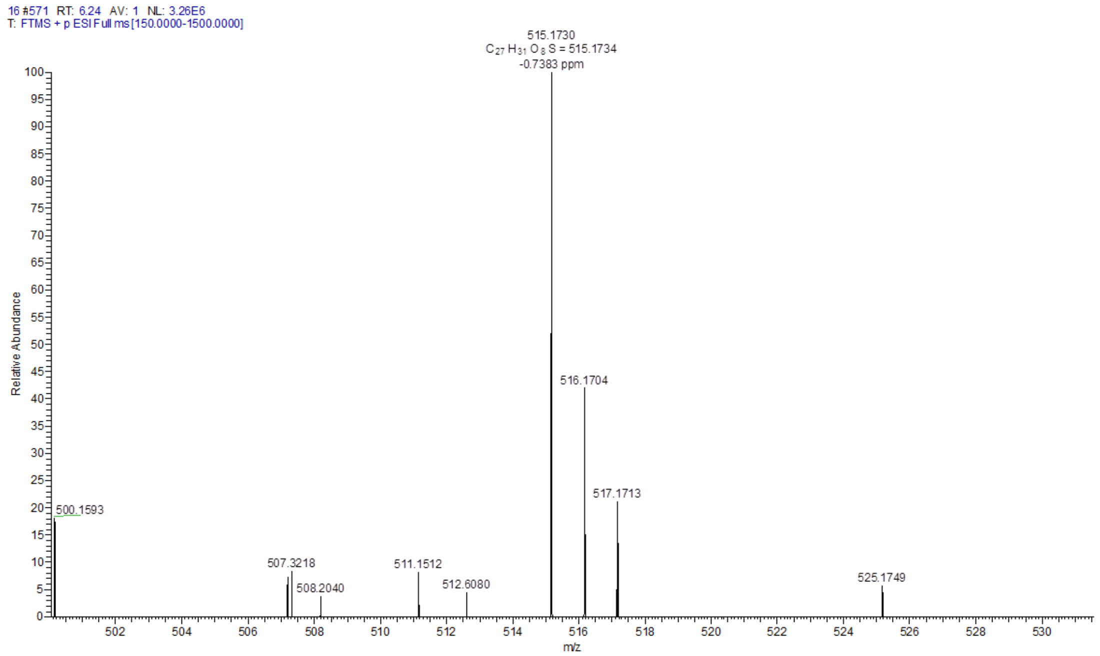

Figure S45. HR-ESI-MS spectrum of compound **37**.

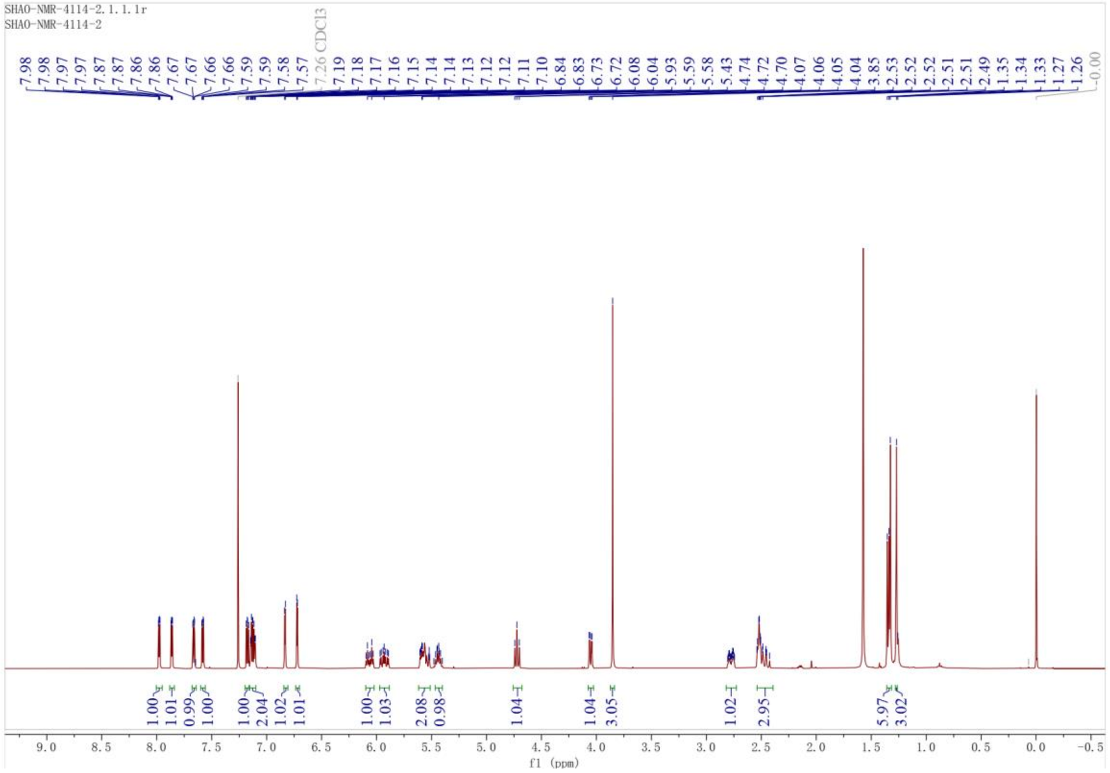

Figure S46. ^{1}H NMR (400 MHz, Chloroform-*d*) spectrum of compound **38**.

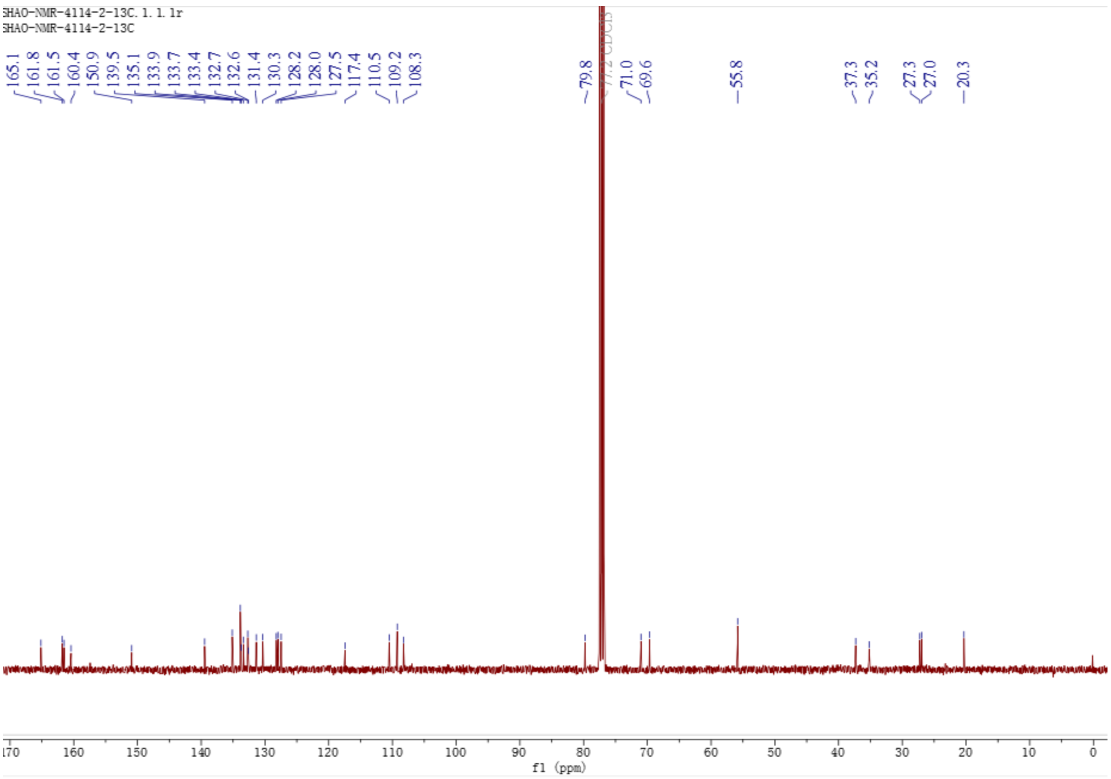

Figure S47. ^{13}C NMR (100 MHz, Chloroform-*d*) spectrum of compound **38**.

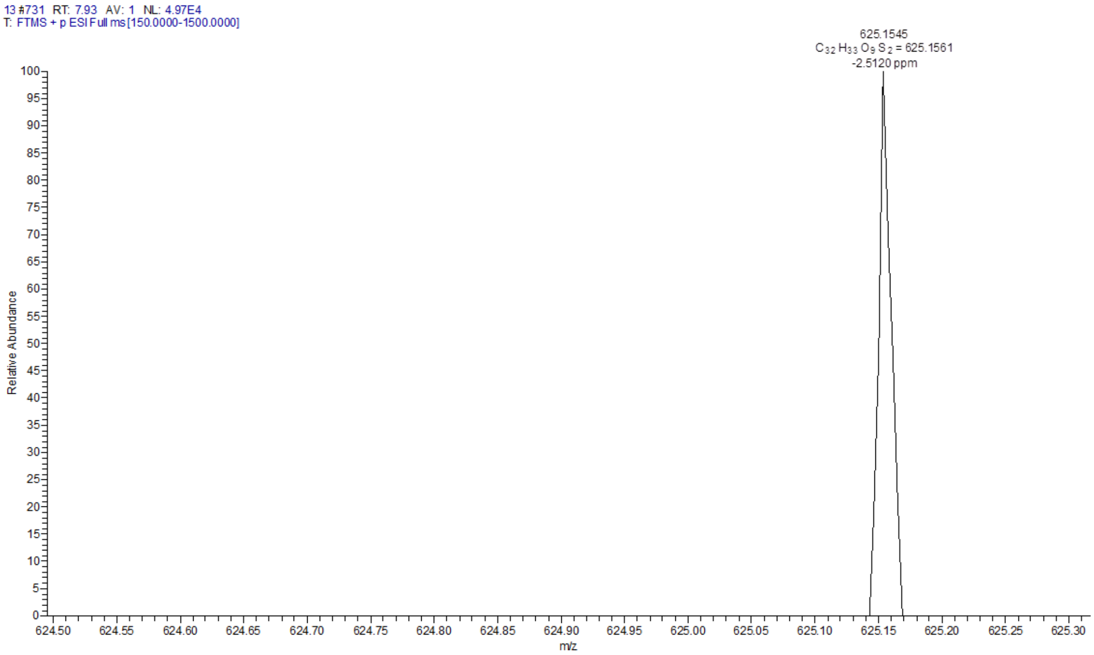

Figure S48. HR-ESI-MS spectrum of compound **38**.
